# Genome Analysis of the Biotechnologically Relevant Acidophilic Iron Oxidising Strain JA12 Indicates Phylogenetic and Metabolic Diversity within the Novel Genus “*Ferrovum*”

**DOI:** 10.1371/journal.pone.0146832

**Published:** 2016-01-25

**Authors:** Sophie R. Ullrich, Anja Poehlein, Judith S. Tischler, Carolina González, Francisco J. Ossandon, Rolf Daniel, David S. Holmes, Michael Schlömann, Martin Mühling

**Affiliations:** 1 Institute of Biological Sciences, TU Bergakademie Freiberg, Leipziger Straße 29, Freiberg, Germany; 2 Georg-August-University Göttingen, Genomic and Applied Microbiology & Göttingen Genomics Laboratory, Grisebachstraße 8, Göttingen, Germany; 3 Center for System Biotechnology, Bio-Computing Division and Applied Genetics Division, Fraunhofer Chile Research Foundation, Avenida Mariano Sánchez Fontecilla 310, Santiago, Chile, and Center for Bioinformatics and Genome Biology, Fundación Ciencia y Vida, Zañartu 1482, and Facultad de Ciencias Biologicas, Universidad Andres Bello, Avenida Los Leones 745, Santiago, Chile; 4 Center for Bioinformatics and Genome Biology, Fundación Ciencia y Vida, Zañartu 1482 and Facultad de Ciencias Biologicas, Universidad Andres Bello, Avenida Los Leones 745, Santiago, Chile; University of Alberta, CANADA

## Abstract

**Background:**

Members of the genus “*Ferrovum*” are ubiquitously distributed in acid mine drainage (AMD) waters which are characterised by their high metal and sulfate loads. So far isolation and microbiological characterisation have only been successful for the designated type strain “*Ferrovum myxofaciens*” P3G. Thus, knowledge about physiological characteristics and the phylogeny of the genus “*Ferrovum*” is extremely scarce.

**Objective:**

In order to access the wider genetic pool of the genus “*Ferrovum*” we sequenced the genome of a “*Ferrovum*”-containing mixed culture and successfully assembled the almost complete genome sequence of the novel “*Ferrovum*” strain JA12.

**Phylogeny and Lifestyle:**

The genome-based phylogenetic analysis indicates that strain JA12 and the type strain represent two distinct “*Ferrovum*” species. “*Ferrovum*” strain JA12 is characterised by an unusually small genome in comparison to the type strain and other iron oxidising bacteria. The prediction of nutrient assimilation pathways suggests that “*Ferrovum*” strain JA12 maintains a chemolithoautotrophic lifestyle utilising carbon dioxide and bicarbonate, ammonium and urea, sulfate, phosphate and ferrous iron as carbon, nitrogen, sulfur, phosphorous and energy sources, respectively.

**Unique Metabolic Features:**

The potential utilisation of urea by “*Ferrovum*” strain JA12 is moreover remarkable since it may furthermore represent a strategy among extreme acidophiles to cope with the acidic environment. Unlike other acidophilic chemolithoautotrophs “*Ferrovum*” strain JA12 exhibits a complete tricarboxylic acid cycle, a metabolic feature shared with the closer related neutrophilic iron oxidisers among the *Betaproteobacteria* including *Sideroxydans lithotrophicus* and *Thiobacillus denitrificans*. Furthermore, the absence of characteristic redox proteins involved in iron oxidation in the well-studied acidophiles *Acidithiobacillus ferrooxidans* (rusticyanin) and *Acidithiobacillus ferrivorans* (iron oxidase) indicates the existence of a modified pathway in “*Ferrovum*” strain JA12. Therefore, the results of the present study extend our understanding of the genus “*Ferrovum*” and provide a comprehensive framework for future comparative genome and metagenome studies.

## Background

Acid mine drainage (AMD) waters are characterised by their acidic pH (< 4) and their high sulfate and metal loads. They are generated due to exposure of metal sulfides in the mined ores, coal or lignite to abiotic and biotic oxidation processes (i.e. [1–3]). These acidic water bodies provide an ecological niche for acidophilic iron and sulfur oxidisers, such as *Acidithiobacillus* spp. and *Leptospirillum* spp., and for acidophilic heterotrophs like *Acidiphilium* spp. [4–7].

In the last decade members of the novel proposed betaproteobacterial genus “*Ferrovum*” [3] have been detected in AMD habitats worldwide [8–18] where they are thought to be involved in iron cycling [7, 13, 17]. Apparently, “*Ferrovum*”-related iron oxidisers prefer higher pH values and ferrous iron concentrations than the well-studied *A*. *ferrooxidans* [15]. The comparison of the microbial composition of the AMD springs Lower and Upper Red Eyes in the Appalachian Mountains (Pennsylvania, USA) revealed the abundant occurrence of *A*. *ferrooxidans* in AMD characterised by pH values below 2.7 and ferrous iron concentrations below 5 mM, while “*Ferrovum*” strains were abundant at pH values around 3 and higher ferrous iron concentrations [15]. These observations are in accordance with studies of other AMD habitats including pit lakes in the Iberian Pyrite Belt (Spain, [13]) and the pilot plant Tzschelln for the biological remediation of AMD which is located close to the lignite mining site Nochten (Lusatia, Germany, [19]. In the context of the latter, it is important to note that apart from contributing to the formation of AMD “*Ferrovum*” has also proved to be useful in a number of biotechnological applications which, apart from the remediation of AMD (e.g. the pilot plant Tzschelln, [8, 19], continuous flow reactor system, [20]), also include the generation of ferric iron rich solutions for the purpose of indirect mineral oxidation [21] and the production of the ferric iron oxyhydroxysulfate schwertmannite which can be used as pigment or chemical absorbent [22].

However, despite their ubiquitous distribution and potential biotechnological relevance the isolation and subsequent physiological characterisation has so far only been successful for the designated type strain “*Ferrovum myxofaciens*” P3G [23]. The isolation of chemolithoautotrophic acidophilic iron oxidisers like “*F*. *myxofaciens*” P3G proved to be demanding, particularly when solid media were used for strain isolation (i.e. [23–27]). These studies also reported a number of reasons for the difficulties related to the isolation, including the contamination of the iron oxidiser with heterotrophic bacteria such as *Acidiphilium* [23, 24, 27] and the high sensitivity of the iron oxidiser to organic compounds released from the agar plates due to acid hydrolysis [25]. Although the development of overlay plates allowed the successful cultivation of many acidophilic bacteria [25, 26], the isolation and culture of novel strains of the genus “*Ferrovum*” has remained an enigma [23] as indicated by the loss of the previously obtained strains “*F*. *myxofaciens*” PSTR [21] and EHS6 [22] prior to a classical microbiological characterisation being conducted. Consequently, the knowledge about the physiological capacities of members of genus “*Ferrovum*” is so far restricted to the physiological characterisation of the type strain “*F*. *myxofaciens*” P3G [23] and to metabolic traits inferred from the draft genome sequence of the type strain [28] and from the metagenomic assembly of the “*Ferrovum*”-like population FKB7 [17].

Metagenomics approaches proved to be extremely valuable to infer metabolic traits of uncultivated members of AMD communities [17, 29–32]. In cases where such a microbial community is either composed of only a few members or where few strains dominate the community, the assembly of even nearly complete genome sequences has been shown to be possible ([29]: *Leptospirillum ferriphilum* and *Ferroplasma acidarmanus*; [32]: *Acidithiobacillus ferrivorans*-like species; [30]: “*Candidatus* Fodinabacter communificans”).

In order to overcome the limitation caused by the extremely difficult isolation of “*Ferrovum*” we sequenced the (meta)genome of the enrichment culture JA12 which represented a mixed culture of a heterotrophic *Acidiphilium* strain and an autotrophic “*Ferrovum*” strain [33]. The mixed culture JA12 originated from samples collected at the pilot plant Tzschelln [8, 19]. Isolation and genome sequence analysis of the contaminating heterotrophic *Acidiphilium* strain JA12-A1 [34] allowed the assembly of the almost complete genome sequence of the novel “*Ferrovum*” strain JA12 from the sequence reads obtained for the mixed culture JA12.

The subsequent in-depth analysis of the genome sequence of “*Ferrovum*” strain JA12 and comparison with other acidophilic iron oxidisers and neutrophilic iron oxidising *Betaproteobacteria* provides (i) the basis for a detailed phylogenetic assignment of “*Ferrovum*” strain JA12, (ii) a comprehensive description of its predicted carbon, nitrogen, sulfur, phosphate and energy metabolism, (iii) information on strategies employed to cope with the chemical constraints of the AMD habitat, and (iv) first insights into metabolic features that distinguish it from the type strain “*F*. *myxofaciens*” P3G.

## Results and Discussion

### Phylogenetic classification of “*Ferrovum*” strain JA12

The iron oxidising mixed culture JA12 was obtained during a previous study [33] from the pilot plant Tzschelln, a mine water treatment plant at the lignite mining site in Nochten (Lusatia, Germany). Geochemical parameters of the water inflow to the pilot plant were reported previously [33]. Ion concentrations and pH were as follows, while phosphate was below the detection limit [35]: Fe^2+^ 270 mg/l, Mn^2+^ 6.3 mg/l, Ni^2+^ 0.081 mg/l, Zn^2+^ 0.18 mg/l, Al^3+^ 1.1 mg/ml, AsO_3_^3-^ 0.031 mg/l, NH_4_^+^ 3.46 mg/l, HCO_3_^-^ 42.1 mg/l, SO_4_^2-^ 1950 mg/l, pH 3.2.

Terminal restriction fragment length polymorphism (TRFLP) and sequence analysis of PCR-amplified 16S rRNA gene fragments revealed that the mixed culture JA12 contained an iron oxidising bacterium related to “*F*. *myxofaciens*” P3G and the heterotrophic alphaproteobacterium *Acidiphilium* sp. JA12-A1 [33]. Since *Acidiphilium* sp. JA12-A1 was successfully grown in pure culture and its genome was sequenced, it was possible to confirm its lack of the ability to oxidise ferrous iron [34]. Thus, it was deduced that the “*F*. *myxofaciens*” P3G-related bacterium, “*Ferrovum*” strain JA12, was responsible for the observed ferrous iron oxidation.

Phylogenetic analyses based on the 16S rRNA gene as molecular marker were performed to evaluate the relationship of strain JA12 to the type strain [23] and to closely related iron oxidisers as well as to non-iron oxidising *Betaproteobacteria* (S1 Fig). As reported previously, the “*F*. *myxofaciens*” P3G-related strains cluster in a distinct branch within the *Betaproteobacteria* [23]. However, the type strain “*F*. *myxofaciens*” P3G and “*Ferrovum*” strain JA12 belong to different subgroups within this distinct branch which is in agreement with the low 16S rRNA gene sequence identity (96%) shared by both strains.

Moreover, the availability of a draft genome sequence of the type strain [28] allowed to infer the level of their phylogenetic relationship *via* three genome sequence-based approaches: the *in silico* estimation of the DNA-DNA hybridisation (DDH) value employing the genome-to-genome distance calculator (GGDC2.0; [36]) as well as, the calculation of the average nucleotide identity based on Blast (ANIb, [37]) and the regression of the tetranucleotide composition (tetra, [38]). A DDH value of 21.6% (formula 2) was estimated for both “*Ferrovum*” genomes with the probability of DDH ≥ 70% being 0%. Their ANIb value was calculated to be 66.04% and the tetra regression value was 0.61. The cut-off values for two distinct species are described to be DDH < 70% [36], ANIb < 95% and tetra regression values < 0.99 [39], respectively. Hence, taken together the 16S rRNA gene sequence identity of less than 98% [40] and the results of the genome-based approaches substantiate the notion that strain JA12 represents a second “*Ferrovum*” species to the type species “*F*. *myxofaciens*”.

### Genome properties

The genome of “*Ferrovum*” strain JA12 currently consists of three contigs with sizes which are 973,749 bp (termed FERRO_contig000001), 962,266 bp (FERRO_contig000002) and 59,722 bp (FERRO_contig000001). The gaps between these contigs are thought to contain ribosomal RNA gene clusters since both ends of FERRO_contig000001 and one end of each of the other two contigs contain ribosomal RNA genes. The therefore nearly complete genome sequence of “*Ferrovum*” strain JA12 consists of 1.99 Mbp and contains 2,001 open reading frames (ORF) of which 1,960 were predicted to be protein-coding genes. The general genome features are summarised in Table 1. The majority of the protein-coding genes (1,462) were assigned to COG classifications (S1 Table).

**Table 1:** Genome properties of “*Ferrovum*” strain JA12.

	Count	Percentage of total [%]
**Number of contigs**	3	
FERRO_contig000001 (size)	973,749 bp	48.79
FERRO_contig000002 (size)	962,266 bp	48.22
FERRO_contig000003 (size)	59,722 bp	2.99
**Total number of bases**	1,995,737	100.00
Gene-coding number of bases	1,850,095	92.70
**G+C content**	887,765	44.48
**Total number of open reading frames**	2,001	100
Protein-coding genes	1,960	97.95
assigned to COGs^1^	1,462	72.77
RNA-coding genes	41	2.05
rRNA	4	0.20
tRNA	36	1.80
other	1	0.05

^1^ Number and percentage of protein-coding genes assigned to the COG categories are shown in S1 Table.

“*Ferrovum*” strain JA12 has an unusually small genome in comparison not only to its closest relatives “*F*. *myxofaciens*” P3G (2.70 Mbp, [28]) and the “*Ferrovum*”-like population FKB7 (2.98 Mbp, [17]), but also to other acidophilic iron oxidising bacteria that typically occur in AMD habitats, such as *A*. *ferrooxidans* ATCC 23270 (2.98 Mbp, [41]) and *Leptospirillum ferrooxidans* C2-3 (2.53 Mbp, [42]) (see also S2 Table). The smaller genome of “*Ferrovum*” strain JA12 may be the result of a genome reduction due to the loss of metabolic functions during its evolution. As explained in further detail below (see section Nutrient assimilation and biomass production) “*Ferrovum*” strain JA12 lacks the gene repertoires involved in nitrogen fixation, sulfur oxidation and utilisation of organic phosphate sources in contrast to other typical members of AMD habitats with larger genomes such as *Acidithiobacillus ferrooxidans* [41]. The loss of these metabolic traits may be the result of an adaptation to the geochemistry of the habitat (pilot plant Tzschelln) or a community adaptive event as described by the Black Queen Hypothesis [43]. The latter states that some members of a microbial community might lose expensive metabolic functions as long as other members of the community compensate for these functions [43, 44]. However, to test these hypotheses in case of “*Ferrovum*” strain JA12 more genomes from other members of the pilot plant community are required. In any case, maintaining a smaller genome may be advantageous for “*Ferrovum*” strain JA12 with respect to a reduced requirement of inorganic phosphate, which often occurs in AMD in very low concentrations [1, 45]. Streamlining of microbial genomes as a means to cope with phosphate limiting growth conditions is well known in oceanic habitats [46].

Apart from the genome size, the G+C content appears to be another distinguishing feature of the two “*Ferrovum*” strains with that of strain JA12 (44.5%) being 10% lower than the G+C content of the type strain (54.9%, [28]).

**Fig 1:**
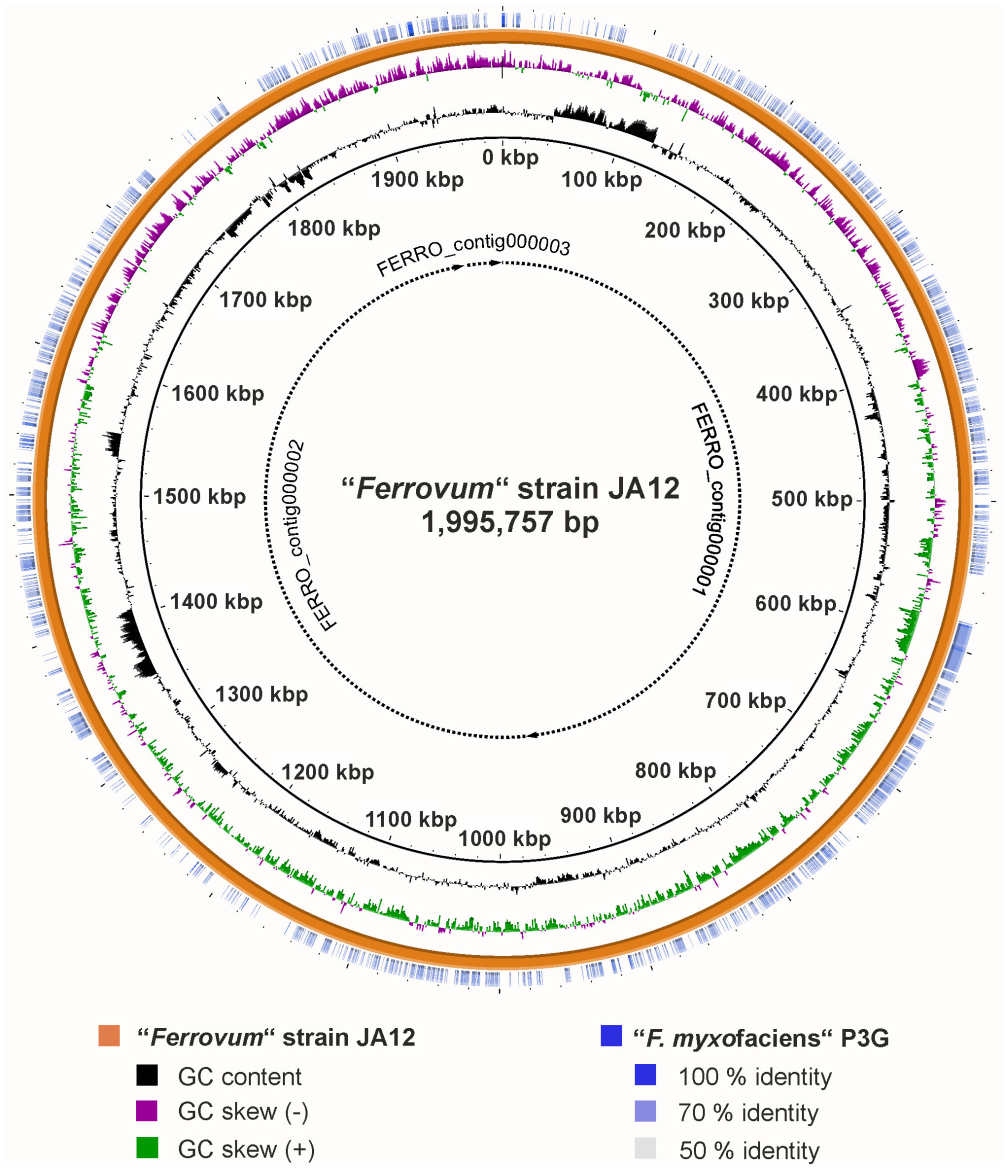
Artificial circular plot of the “*Ferrovum*” strain JA12 genome. The three contigs of “*Ferrovum*” strain JA12 were concatenated to form an artificial circular genome in the order FERRO_contig000001, FERRO_contig000002 and FERRO_contig000003 (broken lines). The origin of replication is not indicated. G+C content and GC skew of the genome of “*Ferrovum*” strain JA12 are shown on ring 1 and 2 from the inside, respectively. The Blastn-based whole genome comparison of “*Ferrovum*” strain JA12 and “*F*. *myxofaciens*” P3G was conducted using BRIG [47] with the genome sequence of “*Ferrovum*” strain JA12 set as reference (ring 3, orange). Blastn matches of “*F*. *myxofaciens*” P3G to the reference are shown on ring 4 (blue) with the colour intensity indicating the sequence identity of the match.

Moreover, the Blastn-based comparison of the genomes (in form of concatenated contigs) of the strains JA12 and P3G indicated differences in their genetic contents which is likely to result in variations of the metabolic potential of the two strains (Fig 1).

### Nutrient assimilation and biomass production

#### Carbon dioxide fixation

**Fig 2:**
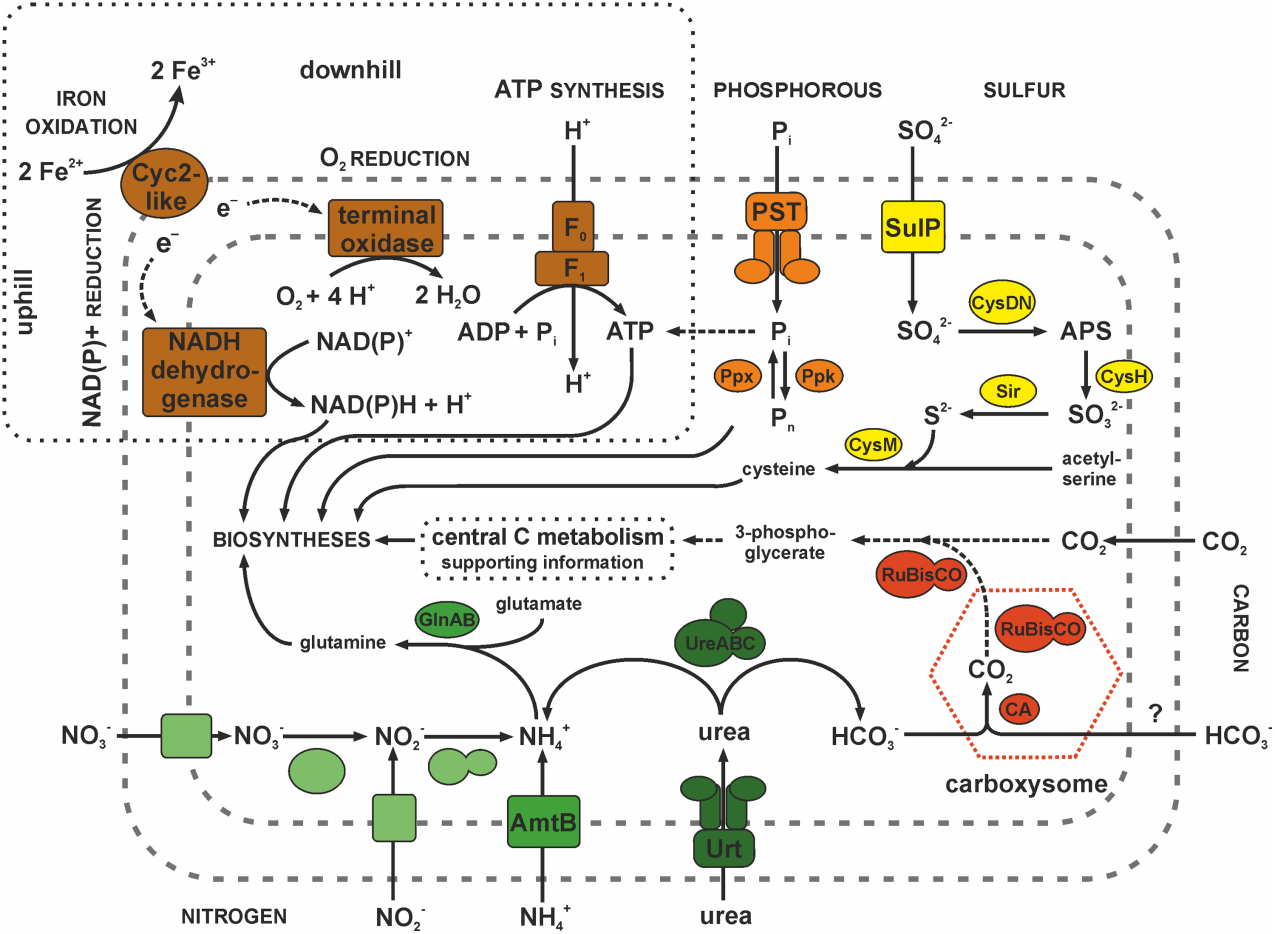
Predicted nutrient assimilation pathways of carbon, nitrogen, phosphorous and sulfur in “*Ferrovum*” strain JA12. Inorganic phosphate is predicted to be taken up by the phosphate specific transport system (PST) and made available to the metabolism or stored as polyphosphate (P_n_). A sulfate permease (SulP) is predicted to be responsible for the uptake of sulfate which then is activated by a sulfate adenylyltransferase to adenosine-phosphosulfate (APS) and subsequently reduced to sulfide. Carbon dioxide appears to be reduced to 3-phosphoglycerate in the Calvin-Benson-Bassham cycle indicated by its key enzyme RuBisCO. Bicarbonate may be fixed in the carboxysome using a carbonic anhydrase (CA) and a RuBisCO. “*Ferrovum*” strain JA12 is predicted to either take up ammonium directly by the ammonium transporter (AmtB) or reduce nitrate or nitrite to ammonium. No names for the nitrate and nitrite transporters and reductases are indicated due to contradicting nomenclature in the databases. Apparently, urea is taken up *via* an ABC transporter (Urt) and hydrolysed to ammonia and bicarbonate by the urease (UreABC). The subsequent spontaneous protonation of ammonia to ammonium at circum neutral pH reduces the proton concentration within the cytoplasm. The energy for all metabolic processes seems to be derived from the oxidation of ferrous iron.

The mixed culture JA12 has successfully been cultivated under autotrophic conditions [33]. Based on its genome sequence, “*Ferrovum*” strain JA12 is able to fix carbon dioxide *via* the Calvin-Benson-Bassham (CBB) cycle (Fig 2 and S3A Table).

The genome contains two copies of each, *cbbS* and *cbbL*, encoding the small and the large subunit of the ribulose-1,5-bisphosphate carboxylase/oxygenase (RuBisCO), respectively. One set consisting of *cbbS* and *cbbL* (FERRO_14010, FERRO_14020) is located in a cluster with genes encoding other CBB cycle enzymes, such as the phosphoribulokinase or the transketolase. The second set (FERRO_08230, FERRO_08240) was detected within a cluster of genes (FERRO_08250—FERRO_08360) predicted to encode carboxysome shell proteins and the carboxysome carbonic anhydrase. The presence of genes involved in the carboxysome formation has also been described for the type strain [28]. The carboxysome is a carbon concentrating mechanism improving the efficiency of carbon dioxide fixation by the RuBisCO and allowing the utilisation of bicarbonate as carbon source [48].

In contrast to the iron oxidising chemolithoautophs *Thiobacillus denitrificans* ATCC 25259 [49] and *Acidithiobacillus ferrooxidans* ATCC 23270 [41, 50], which harbour copies of RuBisCO isotype I and II, both RuBisCOs in “*Ferrovum*” strain JA12 were predicted to be isoform I enzymes. Isoform II RuBisCO is also absent in the type strain [23]. In *A*. *ferrooxidans* ATCC 23270 [51] and *T*. *denitrificans* ATCC 25259 [49] the two isoforms were found to be differentially expressed depending on the oxygen conditions and the electron donor (ferrous iron or reduced sulfur compounds) suggesting that isoform II is only produced under anaerobic conditions. So far anaerobic growth has neither been observed in “*F*. *myxofaciens*” P3G [23] nor in “*Ferrovum*” strain JA12 (unpublished results) which may explain the lack of the RuBisCO isoform II.

In summary, its genetic repertoire allows “*Ferrovum*” strain JA12 to maintain an autotrophic lifestyle with the inorganic carbon sources available in the pilot plant Tzschelln [35]. The primary product of the carbon fixation *via* the CBB cycle is 3-phosphoglycerate which is predicted to be further metabolised in the pathways of the central carbon metabolism in order to generate precursors of bacterial biomass polymers.

#### Central carbon metabolism

The pathways of the central carbon metabolism predicted to be involved in the formation of amino acids, fatty acids, nucleic acids and nucleotide-activated monosaccharides in “*Ferrovum*” strain JA12 are shown in S2A Fig and the corresponding genes are listed in S3A Table. Selected aspects of its central carbon metabolism are outlined in the following.

The tricarboxylic acid (TCA) cycle is a central pathway in the cellular metabolism connecting various pathways with each other. The repertoire of TCA cycle enzymes in an organism has been discussed as indicative for its lifestyle [52]. Comparative genome studies revealed that obligate chemolithoautotrophic prokaryotes are characterised by incomplete TCA cycles lacking the enzyme 2-oxoglutarate dehydrogenase [53]. Whereas *A*. *ferrooxidans* ATCC 23270 [41] and other chemolithoautotrophic acidophiles [53] have been reported to lack the 2-oxoglutarate dehydrogenase, the genomes of the type strain [28] and “*Ferrovum*” strain JA12 were predicted to encode all enzymes of the TCA cycle including the 2-oxoglutarate dehydrogenase (S3A Table). Consequently, the presence of the 2-oxoglutarate dehydrogenase in the type strain has been discussed as the basis of a facultative heterotrophic lifestyle [28], though experimental evidence indicated otherwise [23]. Similarly, the neutrophilic iron oxidisers among the *Betaproteobacteria* (e.g. *S*. *lithotrophicus* ES-1, *G*. *capsiferriformans* ES-2 [54] or *T*. *denitrificans* ATCC 25259 [49]) are obligate chemolithoautotrophs and also harbour the complete TCA cycle.

Moreover, the small repertoire of predicted uptake systems for organic carbon compounds further supports the assumption that “*Ferrovum*” strain JA12 uses carbon dioxide as carbon source. Its genome harbours a putative urea ABC transporter, five putative ABC transport systems for (branched-chain or hydrophobic) amino acids and a potential ABC transporter for di- and oligopeptides (S3B Table). However, permeases for amino acids as predicted in *A*. *ferrooxidans* ATCC 23270 [41] and uptake systems for mono- or disaccharides as described in *A*. *caldus* SM-1 [55] were not detected in the genome of “*Ferrovum*” strain JA12. The identified amino acid and peptide transporters in “*Ferrovum*” strain JA12 may be involved in the recycling of peptides derived from the turnover processes of the cell envelope as it was shown for many other bacteria [56].

Since the massive formation of extracellular polymeric substances (EPS) in particular exopolysaccharides represents the most prominent phenotypic feature of the type strain [9, 23, 57], we focussed our further analyses on the identification of genes putatively involved in the synthesis of exopolysaccharides. In this context it should be clarified that “*Ferrovum*” strain JA12 has not been observed to produce comparable amounts of EPS. However, this observation may be due to the fact that “*Ferrovum*” strain JA12 is usually cultivated at pH 2.8 to 3.0, a pH value at which the formation of the ferric iron oxyhydroxy sulfate mineral schwertmannite occurs in close proximity to the iron oxidising cells [22]. Consequently, the formation of EPS would be difficult to detect in “*Ferrovum*” strain JA12. Nevertheless, we identified genes encoding enzymes of the nucleotide sugar metabolism that are predicted to be involved in the formation of dTDP-rhamnose, UDP-glucose, UDP-galactose, UDP-glucuronic acid, UDP-galacturonic acid and UDP-mannose, all of which may serve as precursors for the synthesis of exopolysaccharides. These genes are organised in a gene cluster (FERRO_01000—FERRO_01150) similar to the gene cluster reported to be involved in the formation of exopolysaccharides in *A*. *ferrooxidans* ATCC 23270 [58]. The potential of “*Ferrovum*” strain JA12 to produce exopolysaccharides is also supported by the high number of glycosyltransferases encoded by its genome (S3A Table and in S2B Fig).

Moreover, genes encoding an acetyl-CoA acetyltransferase, an acetoacetyl-CoA reductase and a poly(R)-hydroxyalkanoic acid synthase presumably enable “*Ferrovum*” strain JA12 to synthesise polyhydroxybutyrate (PHB) from acetyl-CoA. The “*Ferrovum*” strain JA12 genome also contains two copies of a gene predicted to encode a polyhydroxyalkanoate depolymerase which catalyses the hydrolysis of PHB (S3A Table and S2A Fig). PHB serves as organic carbon storage compound under growth limiting conditions in many bacteria [59, 60].

#### Nitrogen

Based on its gene repertoire, “*Ferrovum*” strain JA12 appears to be able to assimilate nitrogen from various sources including ammonium, nitrate and urea (Fig 2 and S3B Table) [61]. But unlike the type strain “*F*. *myxofaciens*” P3G [23], “*Ferrovum*” strain JA12 does not fix molecular nitrogen by the standard pathway since its genome does not encode the nitrogenase nor any other *nif*-genes.

We hypothesise that “*Ferrovum*” strain JA12 can take up ammonium by an ammonium transporter of the AmtB family and incorporate it to glutamate using a glutamine synthetase, thereby making it available for the production of biomass.

The identification of a gene cluster (FERRO_17980—FERRO_18020) predicted to encode transporters and reductases for nitrate and nitrite indicates the potential of “*Ferrovum*” strain JA12 to reduce nitrate. Since the nitrate reductase appeared to lack transmembrane helices and because the gene cluster in which it is embedded lacks the other subunits of the membrane-bound respiratory nitrate reductase complex [62, 63], we propose that nitrate does not serve as alternative terminal electron acceptor. Instead, the predicted nitrate reductase contains the conserved domain occurring in assimilatory nitrate reductases (NCBI: cd02754) and it shares a protein sequence identity of 73% with the assimilatory nitrate reductase of *S*. *lithotrophicus* ES-1 (Slit_0160) [54]. Hence, the gene cluster apparently enables “*Ferrovum*” strain JA12 to reduce nitrate to ammonium and to utilise it as an alternative nitrogen source as has also been predicted for the “*Ferrovum*”-like population FKB7 [17]. This proposed scenario is further supported by the presence of the predicted cytoplasmic assimilatory nitrite reductase in the same gene cluster.

**Fig 3:**
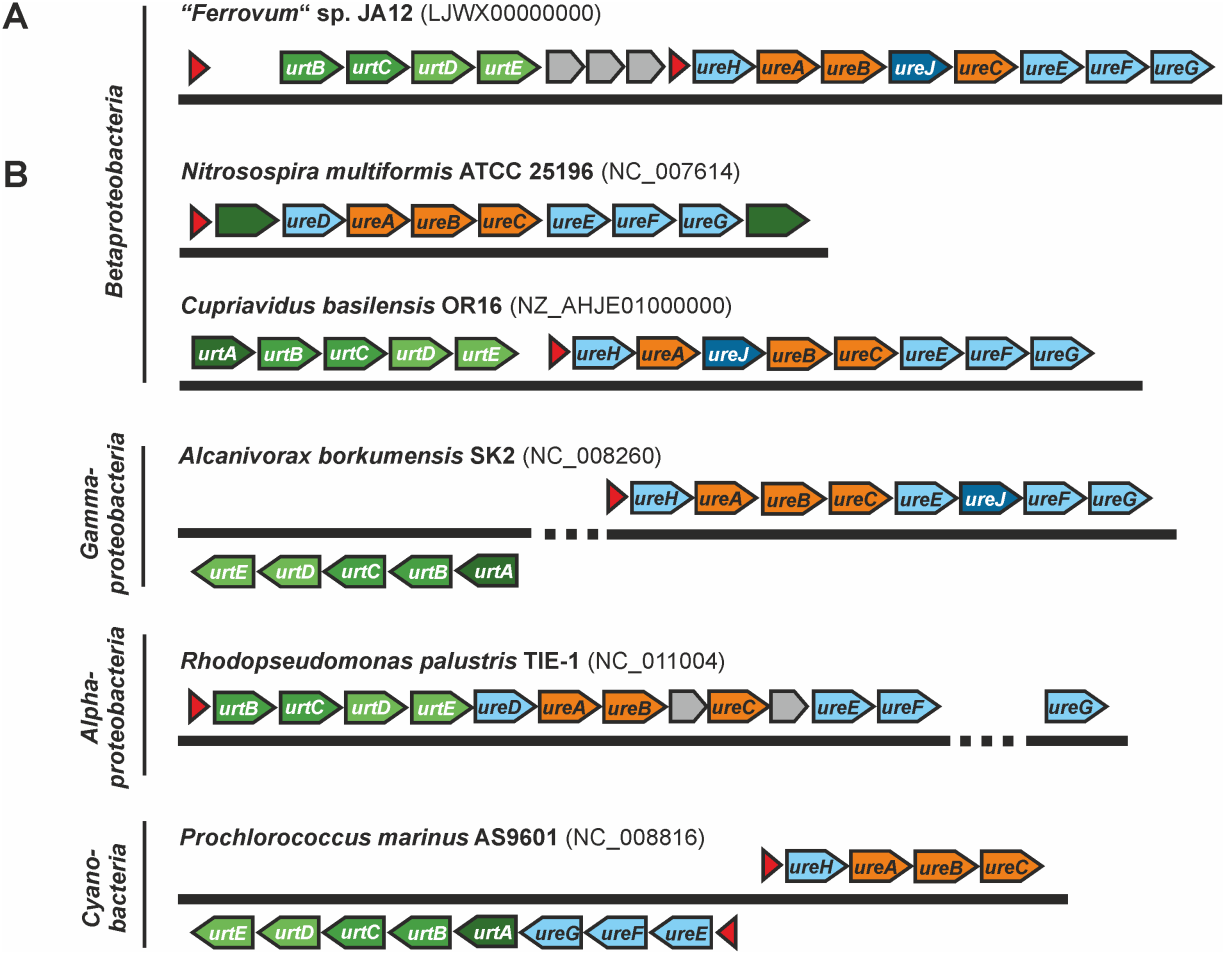
Urease-encoding gene clusters in “*Ferrovum*” strain JA12 and representative bacteria belonging to other taxa. The structure of the urease gene cluster in the genome of “*Ferrovum*” strain JA12 (A) was compared to urease encoding gene clusters of other urease active bacteria (B). With the exception of *Nitrosospira multiformis* ATCC 25196, which appears to use a urea-specific permease, urea is taken up *via* an ABC transport system (green), consisting of a periplasmic urea binding protein (*urtA*), two permeases (*urtBC*) and two ATP-hydrolysing enzymes (*urtDE*). The genes predicted to encode the three urease subunits (*ureABC*, orange), the Ni^2+^ transporter (*ureJ*, dark blue) and the urease accessory proteins (*ureH*, *ureEFG*, light blue) are often co-localised with the urea transporter. The accessory proteins encoded by *ureH* and *ureD* are thought to fulfil the same function during the formation of the active urease. Genes encoding other proteins involved in nitrogen metabolism are shown in grey. The location of potential promotors (red) were predicted using FGENESB.

We also identified a gene cluster that was predicted to encode a putative urea ABC transporter (*urtABCDE*), the urease subunits (*ureABC*) and the urease accessory proteins (*ureH*, *ureJ*, *ureEFG*) (Fig 3A). While *ureJ* encodes a Ni^2+^-uptake transporter, the other urease accessory proteins are predicted to be involved in the assembly of the Ni^2+^-containing metallo-centre of the catalytically active urease. Urease has been shown to hydrolyse urea to ammonia and bicarbonate in a number of organisms [64]. Since ammonia is present as ammonium at cytoplasmic pH (thought to be circum neutral) the urease synthesis may promote the utilisation of urea as an alternative nitrogen source in “*Ferrovum*” strain JA12.

In addition, carbonic anhydrases associated with carboxysomes in other organisms [48] may also facilitate the use of bicarbonate, derived from urea hydrolysis, as a carbon source (Fig 2). Alternatively, urease activity in “*Ferrovum*” strain JA12 may also play an important role in pH homeostasis (see below, section Strategies to adapt to acidic environments, high metal loads and oxidative stress).

The presence and physiological relevance of the urease encoding gene cluster is known for the phototrophic iron oxidising *Alphaproteobacteria Rhodopseudomonas palustris* [65] and *Rhodobacter capsulatus* [66]. But with exception of the draft genome sequence of the “*Ferrovum*”-like population FBK7 [17], a gene cluster encoding homologous genes has so far neither been detected in any other iron oxidising *Betaproteobacteria* nor in the genomes of any acidophilic iron oxidising *Gammaproteobacteria* or *Acidithiobacillia*. The organisation of the gene cluster in “*Ferrovum*” strain JA12 was highly similar to that of the soil bacterium *Cupriavidus basilensis* OR16 (Fig 3B), which was striking given the fact that the composition and structure of the urease encoding gene clusters appeared to be in general not conserved among the bacteria included in the comparison.

Based on the chemical composition of the inflow to the pilot plant, ammonium presents the main source of nitrogen for “*Ferrovum*” strain JA12 and, thus, the lack of the ability to fix molecular nitrogen may be of no disadvantage. The nitrate and urea content was not determined in the pilot plant water. However, traces of urea in the AMD waters of the lignite mining site may be generated by bioweathering processes of the fossil organic matter in the lignite similar to bioweathering of the organic matter in copper shales [67, 68].

#### Phosphate

Seven genes of the *pho*-regulon were predicted in the “*Ferrovum*” strain JA12 genome (S3C Table). This regulon is widespread and highly conserved among bacteria [69] and it presumably enables “*Ferrovum*” strain JA12 to take up inorganic phosphate *via* a phosphate specific ABC transporter. We hypothesise that the expression of the transporter genes *pstSABC* is regulated by a system involving a histidine sensor kinase (PhoR), a transcription factor (PhoB) and an inhibitory protein (PhoU) as has been described in other organisms [69]. Inorganic phosphate may be stored as polyphosphate involving a polyphosphate kinase and an exopolyphosphatase for the synthesis and hydrolysis of polyphosphates, respectively (Fig 2).

Inorganic phosphate was not detectable in the pilot plant Tzschelln [35] suggesting that its concentration was very low and may be growth limiting. Hence, the ability to utilise organic phosphate sources such as phosphonates as reported for *A*. *ferrooxidans* ATCC 23270 [41] should be advantageous. However, genes predicted to encode specific ABC transporters and C-P-lyases (i.e. *phn*-genes) for the uptake and cleavage of phosphonates, respectively [69], were not detected in the genome of “*Ferrovum*” strain JA12 and they were also absent in the type strain. Other community members in the pilot plant may provide phosphate *via* the utilisation of phosphonates, as would be suggested by the Black Queen hypothesis ([43]). This may therefore represent a similar scenario to the generation of fixed nitrogen sources by a small fraction of community members that has been reported for other AMD habitats [7, 29].

#### Sulfate

Sulfate represents one of the most abundant ion species in the inflow to the pilot plant Tzschelln [35]. It appears to serve as sulfur source for “*Ferrovum*” strain JA12. Analysis of the genome of “*Ferrovum*” strain JA12 suggests the uptake of sulfate by a sulfate permease of the SulP-type (FERRO_00170) and the assimilation *via* the adenosine phosphosulfate (APS) pathway as has also been predicted for *A*. *ferrooxidans* ATCC 23270 [70] (Fig 2 and S3D Table). First, sulfate could be activated to APS by the sulfate adenylyltransferase and subsequently reduced to sulfite by the APS reductase. Sulfite is predicted to be further reduced to sulfide by a ferredoxin-dependent sulfite reductase encoded by *sir* (FERRO_11530) unlike *A*. *ferrooxidans* that is predicted to reduce sulfite using a NADH-dependent sulfite reductase encoded by *cysJI* [70]. A gene homologous to *sir* was also detected in the genomes of the type strain, *S*. *lithotrophicus* ES-1, *T*. *denitrificans* ATCC 25259 and *G*. *capsiferriformans* ES-2. Finally, sulfide may be transferred to acetyl serine to form cysteine catalysed by the enzyme cysteine synthase. Acetyl serine is predicted to be generated by the serine O-acetyltransferase.

However, in contrast to *A*. *ferrooxidans* ATCC 23270 [41] the genome of “*Ferrovum*” strain JA12 seems not to encode an APS kinase for the alternative activation of sulfate to phosphoadenosine phosphosulfate (PAPS) and subsequent reduction to sulfide *via* the PAPS pathway.

### Energy metabolism

#### Ferrous iron oxidation

Both “*Ferrovum*” strain JA12 and the type strain appear to gain the energy for all metabolic processes and biomass production from the aerobic oxidation of ferrous iron. Predicted redox proteins in “*Ferrovum*” strain JA12 were compared to redox proteins known to be involved in the iron oxidation in other prokaryotes (Table 2) placing special emphasis on the well-studied processes in *A*. *ferrooxidans*.

**Table 2:** Redox proteins of the ferrous iron oxidation in “*Ferrovum*” strain JA12 and other iron oxidisers.

		Potential redox proteins		
Class	Species	Outer membrane	Periplasm	Inner membrane
*Alphaproteo-bacteria*	*Rhodopseudomonas palustris* TIE-1 (neutro.)^a^	PioB, PioA	PioA	?
	*Rhodobacter capsulatus* SB1003 (neutro.)^b^		Cytochrome *c* (FoxE), pyrroloquinoline quinone containing protein (FoxY)	inner membrane protein (FoxZ)
*Betaproteo-bacteria*	*Gallionella capsiferriformans* ES-2 (neutro.)	PioB, PioA	PioA	?
	*Sideroxydans lithotrophicus* ES-1 (neutro.) ^c^	?	Fe-S molybdopterin oxidoreductase, cytochrome *c*	Cytochrome *c* oxidase *cbb*_3_-type; cytochrome *bd* complex
	*“Ferrovum myxofaciens”* P3G (acido.)^d^	Cyc2-like	Cupredoxin, cytochromes *c*	Cytochrome *c* oxidase *cbb*_3_-type
	*“Ferrovum*-like FKB7 (acido.)^e^	Cyc2-like; Iro-like	Cyc1-like, ?	?
	“*Ferrovum*” strain JA12 (acido.)^f^	Cyc2-like	Cytochrome *c*	Cytochrome *c* oxidase *cbb*_3_-type; cytochrome *bo*_3_ ubiquinol oxidase; cytochrome *bd* complex
*Acidithio-bacillia*	*Acidithiobacillus ferrooxidans* ATCC 23270 (acido.) ^g^	Cyc2	Rusticyanin A/B, Cyc1	Cytochrome *c* oxidase *aa*_3_-type
	*Acidithiobacillus ferrivorans* SS3 (acido.) ^h^		Iro, rusticyanin A/B, Cyc1	Cytochrome *c* oxidase *aa*_3_-type
*Gammaproteo-bacteria*	*Thiobacillus prosperus* V6 (acido.) ^i^	Cyc2-like	Rusticyanin-like, ?	Cytochrome *c* oxidase *aa*_3_-type
*Zeta-proteobacteria*	*Mariprofundus ferrooxydans* PV-1 (neutro.) ^j^	?	Fe-S molybdopterin oxidoreductase, cytochrome *c*	Cytochrome *c* oxidase *cbb*_3_-type; cytochrome *bd* complex
*Nitrospira*	*Leptospirillum* spp. (acido.) ^k^	Cyc_572_	Cyc_579_, cytochrome *c*	Cytochrome *c* oxidase *cbb*_3_-type
*Thermopotei*	*Sulfolobus metallicus* (acido.) ^l^			CbsA-like cytochrome *b*, haem-Copper terminal oxidase
*Thermo-plasmata*	*Ferroplasma* spp. (acido.) ^m^	?		?, sulfocyanin, Cytochrome *c* oxidase *cbb*_3_-type

Redox proteins are assigned to their (postulated) localisation (based on [71, 72]). Proteins presumably required but not yet identified are indicated by question mark. The lifestyle of the species is indicated in parentheses: acido., acidophilic; neutro., neutrophilic. The encoding genes for “*Ferrovum*” strain JA12 are listed in Table E in S3 Table.;

^a^ [73];

^b^ [74];

^c^ [75];

^d^ [28];

^e^ [17];

^f^ [61], this publication;

^g^ [76–78];

^h^ [79, 80];

^i^ [78, 81];

^j^ [82];

^k^ [29];

^l^ [83];

^m^ [84].

**Fig 4:**
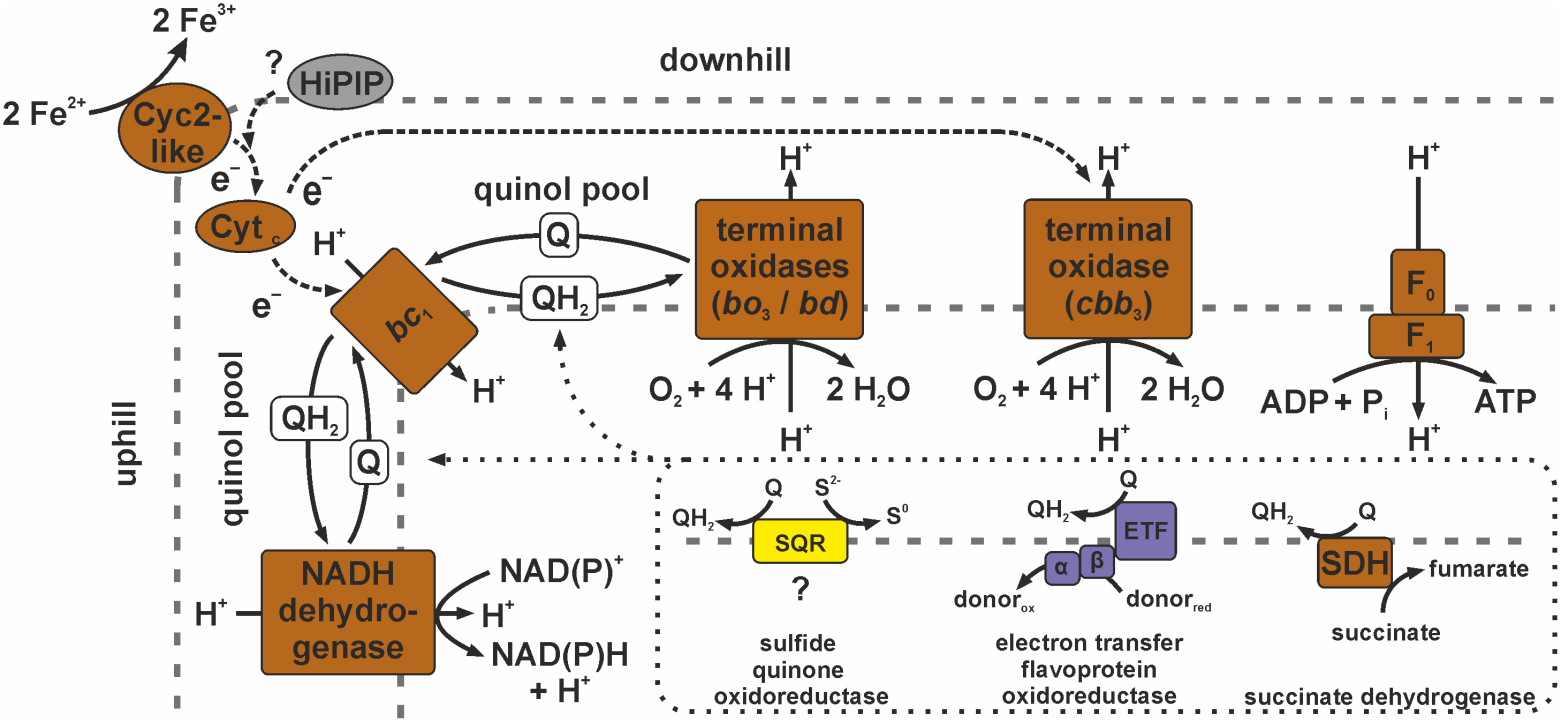
Predicted electron transfer from ferrous iron to the terminal electron acceptors in “*Ferrovum*” strain JA12. Ferrous iron is probably oxidised at the cell surface by a high molecular mass cytochrome (Cyc2-like). The relevance of the identified high potential iron-sulfur protein (HiPIP) in this process remains unknown. The electrons appear to be further transferred *via* soluble *c*-type cytochromes in the periplasm either uphill driven by the proton motive force to the *bc*_1_ complex and NADH dehydrogenase to produce reduction equivalents (NAD(P)H), or downhill *via* the *cbb*_3_-type cytochrome *c* oxidase or alternatively *via* one of the quinol oxidases (cytochrome *bd* complex, cytochrome *bo*_3_ ubiquinol oxidase) to the terminal acceptor oxygen. The reduction of oxygen to water at the terminal oxidases neutralises protons entering the cell during ATP synthesis *via* the ATP synthase (F_0_F_1_). The terminal oxidases *bo*_3_ and *cbb*_3_ pump protons into the periplasm driven by the downhill electron transfer to oxygen. Since the identity of the quinol derivates produced by “*Ferrovum*” strain JA12 was not investigated QH_2_ represents the reduced form and Q the oxidised form of the quinol derivative, respectively. The genome also encodes other potential oxidoreductases which could channel electrons into the quinol pool (e.g. sulfide quinone oxidoreductase (SQR), electron transfer flavoprotein oxidoreductase (ETF), succinate dehydrogenase (SDH) of the citrate cycle).

In *A*. *ferrooxidans* ferrous iron is oxidised in the outer membrane *via* the high molecular mass cytochrome Cyc2 [41, 76]. The small copper protein rusticyanin transfers electrons from Cyc2 either downhill *via* Cyc1 to the *aa*_3_-type terminal oxidase, where reduction of oxygen takes place, or uphill *via* CycA-1 and the *bc*_1_ complex to the NADH-quinone oxidoreductase complex [41, 77, 85]. The inferred model of the potential electron transfer processes in “*Ferrovum*” strain JA12 is shown in Fig 4 (see also S3E Table).

The genome of “*Ferrovum*” strain JA12 was predicted to encode a high molecular mass cytochrome (Cyc2-like; FERRO_13930) that had a sequence identity of 29% to the homologous protein Cyc2 of *A*. *ferrooxidans* ATCC 23270 (AFE_3153). Furthermore, the similar pattern of hydrophobic transmembrane helices and hydrophilic regions of both proteins (S3A Fig) suggests a similar localisation of the Cyc2-like protein in the outer membrane of “*Ferrovum*” strain JA12 [41, 76]. The type strain was also thought to involve a Cyc2-like cytochrome in the ferrous iron oxidation [28].

However, an alternative iron oxidation reaction was proposed for the “*Ferrovum*”-like population FKB7 based on the draft genome sequence assembled from metagenomics reads [17]. FKB7 was predicted to oxidise ferrous iron *via* the iron oxidase-like high potential iron-sulfur protein that shares a sequence identity of 26% to the iron oxidase Iro of *A*. *ferrivorans* SS3 (Acife_3266) and of 32% to high potential iron-sulfur protein Hip in *A*. *ferrooxidans* ATCC 23270 (AFE_2732). While Iro has been shown to be involved the ferrous iron oxidation, Hip is more likely involved in the oxidation of reduced sulfur compounds [77, 79, 85]. The genome of “*Ferrovum*” strain JA12 was predicted to encode a homologous high potential iron-sulfur protein (FERRO_02670) which shares 71% sequence identity with the iron oxidase-like protein in FKB7. However, a phylogenetic comparison of the amino acid sequences of the predicted high potential iron-sulfur protein of “*Ferrovum*” strain JA12 (FERRO_02670) with those of Iro from *A*. *ferrivorans* SS3 and Hip from *A*. *ferrooxidans* ATCC 23270 did not provide supporting evidence for its involvement in the ferrous iron oxidation in “*Ferrovum*” strain JA12 as hypothesised for FKB7 (S4A Fig).

Since no homologous protein to rusticyanin of *A*. *ferrooxidans* (AFE_3146) could be detected in the genome of “*Ferrovum*” strain JA12, we hypothesise that the electrons from ferrous iron oxidation may be passed directly from the Cyc2-like cytochrome in the outer membrane *via* soluble *c*-type cytochromes in the periplasm to the respiratory complexes in the inner membrane. The *c*-type cytochromes (CytC, FERRO_02680; CytC, FERRO_02750) were identified using the protein sequences of Cyc1 (AFE_3152) and CycA-1 (AFE_3107) of *A*. *ferrooxidans*. Both were predicted to be soluble proteins in contrast to Cyc1 (S3B Fig). Possibly, one of the *c*-type cytochromes in “*Ferrovum*” strain JA12 transfers the electrons downhill to the terminal oxidase while the other passes the electrons to the uphill branch similar to Cyc1 and CycA-1 in *A*. *ferrooxidans* ATCC 23270 [41, 77, 85]. However, a phylogenetic comparison to Cyc1 and CycA-1 did not allow to assign these specific roles to the *c*-type cytochromes in “*Ferrovum*” strain JA12 (S4B Fig).

The terminal electron acceptor of the downhill branch is oxygen. In contrast to *A*. *ferrooxidans* ATCC 23270, which was reported to use an *aa*_3_-type cytochrome *c* oxidase as terminal oxidase [41, 77, 85], “*Ferrovum*” strain JA12 was predicted to reduce oxygen *via* a *cbb*_3_-type cytochrome *c* oxidase similar to the neutrophilic iron oxidisers *Mariprofundus ferrooxydans* PV-1 [82] and *S*. *lithotrophicus* ES-1 [75] and similar to the acidophiles “*F*. *myxofaciens*” P3G [28] and *Leptospirillum ferriphilum* ML-4 [29]. Although only genes predicted to encode the subunits I and II of the *cbb*_3_-type cytochrome *c* oxidase were identified in the genome of “*Ferrovum*” strain JA12, one of the co-localised *c*-type cytochromes (FERRO_02610, FERRO_02510) could substitute the missing subunit III, which also represents a *c*-type cytochrome [86] (S4C Fig).

Furthermore, two alternative terminal oxidases were predicted that use quinol as electron donor instead of the soluble *c*-type cytochromes: the cytochrome *bo*_3_ ubiquinol oxidase and the cytochrome *bd* complex.

“*Ferrovum*” strain JA12 presumably generates reduction equivalents for biosyntheses similar to *A*. *ferrooxidans* transferring electrons from the *c*-type cytochrome in the periplasm *via* the *bc*_1_ complex and the quinol pool to the NADH-quinone oxidoreductase complex [41, 77, 85]. In acidophilic iron oxidisers this uphill electron transfer from ferrous iron to NAD(P)^+^ is driven by the proton motive force which is provided by the natural proton gradient between the environment and the cytoplasm [78, 87, 88].

#### Other redox reactions connected to the quinol pool

The genome analysis revealed further electron transferring proteins that may channel electrons into the quinol pool including the predicted succinate dehydrogenase as well as a predicted electron transfer flavoprotein (ETF) and a predicted flavoprotein dehydrogenase (ETF ubiquinone oxidoreductase) (Fig 4 and S3E Table).

Based on the protein sequence analysis no specific function could be inferred for the ETF and its dehydrogenase in “*Ferrovum*” strain JA12. However, the co-localisation of the ETF subunits encoding genes with genes predicted to encode an acetyl-CoA acyltransferase (FERRO_16660) and a 3-hydroxyacyl-CoA dehydrogenase (FERRO_16670) may indicate an involvement in the fatty acid metabolism.

Furthermore, the genes putatively encoding a sulfide:quinone oxidoreductase and a rhodanese-like protein were detected in the direct neighbourhood of the cytochrome *bd* complex encoding genes. The sulfide:quinone oxidoreductase and the rhodanese-like protein have been shown to be involved in the oxidation of reduced sulfur compounds in *A*. *ferrooxidans* ATCC 23270 [78]. Due to the lack of a pure culture of “*Ferrovum*” strain JA12 we could not test its ability to oxidise reduced sulfur compounds. However, the genome of “*Ferrovum*” strain JA12 seems to lack genes encoding other proteins that are additionally required for the oxidation of sulfur compounds in *A*. *ferrooxidans* (*hdr*, heterodisulfide reductase; *sreABCD*, sulfur reductase [89]) and in *T*. *denitrificans* (*sox*-operon, sulfur oxidation; *dsr*-operon, dissimilatory sulfate reduction; [49]). Taking furthermore into account, that the type strain was shown to be unable to use sulfur compounds as electron donor [23] despite encoding a putative sulfide:quinone oxidoreductase, it seems unlikely that “*Ferrovum*” strain JA12 uses sulfur compounds as electron donor.

#### Predicted formate dehydrogenase

The genome analysis of “*Ferrovum*” strain JA12 also revealed a cluster of genes predicted to encode the β-, α-, and δ-subunits of a formate dehydrogenase H and a formate dehydrogenase accessory protein (S3E Table). These genes, which are adjacent to a transposase (FERRO_08080), were not detected in the genome of the type strain.

The lack of any hydrogenase-encoding genes in “*Ferrovum*” strain JA12 suggested its inability to form the hydrogen-evolving formate hydrogenlyase complex as described for *A*. *ferrooxidans* ATCC 23270 [41]. Thus, the formate dehydrogenase in “*Ferrovum*” strain JA12 is presumed to play a different yet unknown role.

According to a Blastp search against the NCBI non-redundant database the best hit for the catalytically active α-subunit of the “*Ferrovum*” strain JA12 enzyme (FERRO_08040) was the homologous protein of *Pseudogulbenkiania ferrooxidans* 2002 (FuraDRAFT_2186). Both proteins share a sequence similarity of 79% and their encoding genes are located in similarly structured gene clusters. Since *P*. *ferrooxidans* is a nitrate-dependent iron oxidiser [90] its formate dehydrogenase may be involved in the nitrate respiration potentially delivering electrons *via* the quinol pool as is has been discussed in other bacteria [91, 92]. Although we were not able to test a nitrate-dependent anaerobic growth in “*Ferrovum*” strain JA12, due to the lack of pure culture, a similar scenario for “*Ferrovum*” strain JA12 seems unlikely since no genes predicted to encode the respiratory nitrate reductase were identified in its genome.

### Strategies to adapt to acidic environments, high metal loads and oxidative stress

#### Acidic environment

**Fig 5:**
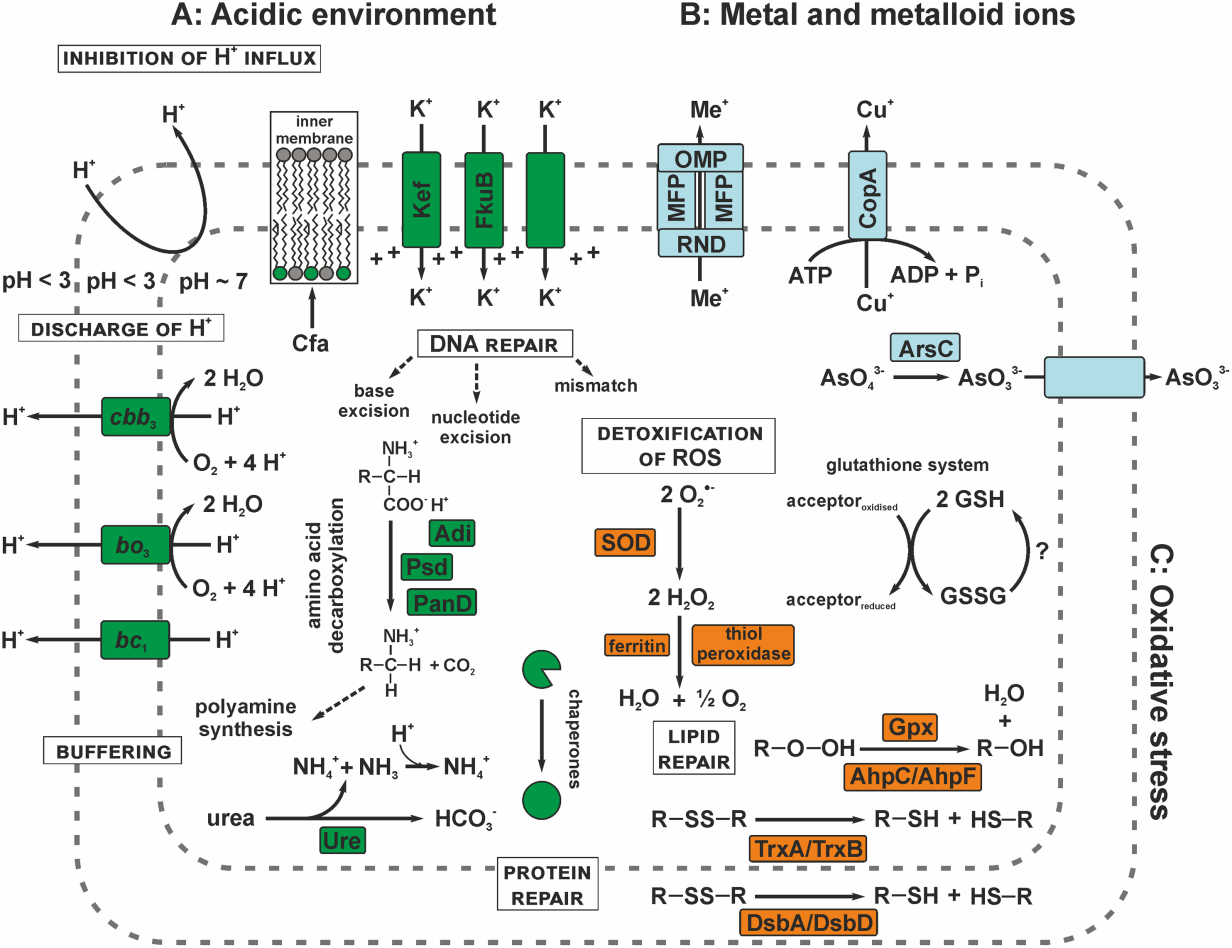
Predicted stress management strategies in “*Ferrovum*” strain JA12. “*Ferrovum*” strain JA12 appears to employ strategies acting at different levels to maintain the intracellular pH homeostasis (A, green). Uncontrolled proton influx could be inhibited by incorporating cyclopropane fatty acyl phospholipids (Cfa) in the membrane or by building up an inside positive membrane potential by the increased uptake of K^+^-ions (Kef, FkuB). Apparently, the complexes of the respiratory chain (*bc*_1_, *bo*_3_, *cbb*_3_) contribute to the active discharge of protons. Furthermore, “*Ferrovum*” strain JA12 is predicted to buffer protons by the decarboxylation of arginine (Adi), phosphatidylserine (Psd) or aspartate (PanD) and by the hydrolysis of urea *via* the urease (Ure). Damaged proteins appear to be restored by a number of chaperones. “*Ferrovum*” strain JA12 could cope with the high concentrations of metal and metalloid ions (B, light blue) either using specific systems like the copper efflux pump (CopA) or arsenate reductase (ArsC) and the arsenite efflux pump. Alternatively, more general multidrug transport systems of the RND family involving an efflux pump (RND), membrane fusions proteins (MFP) and an outer membrane protein (OMP) could be used to extrude metal ions. Oxidative stress (C, orange) is apparently managed by the detoxification of reactive oxygen species (ROS) using a superoxide dismutase (SOD) or thiol peroxidases and ferritin. Damaged proteins could be repaired in the cytoplasm using a thioredoxin (TrxA)/ thioredoxin reductase (TrxB)-dependent system or *via* the thiol:disulfide interchange proteins (DsbA, DsbD) in the periplasm. Damaged lipids may be restored by the peroxiredoxin (AhpC)/ alkyl hydroperoxide reductase (AhpF)-dependent system or by the glutathione peroxidase (Gpx). “*Ferrovum*” strain JA12 could use glutathione (GSH)-dependent systems to restore the redox balance, though it remains unclear how glutathione disulfide (GSSG) is recycled. A wide repertoire of DNA repair systems is predicted serve in the repair of damaged DNA (see also S3F Table).

“*Ferrovum*” strain JA12 appears to employ strategies to maintain the internal cytoplasmic pH that act at various cellular levels as proposed by [93, 94]: (i) the prevention of uncontrolled influx of protons, (ii) the active discharge of protons and buffering of the cytoplasmic pH and (iii) the repair of damages to biomolecules caused by high intracellular proton concentrations (Fig 5A and S3F Table).

The membrane structure is considered to be a very important aspect of blocking proton influx [93, 94]. Bacteria can prevent uncontrolled proton influx by increasing the fraction of saturated fatty acids (*A*. *caldus*: [95]), or by incorporating cyclopropane fatty acids, a strategy that has been found in many neutrophiles [96–98]. In the “*Ferrovum*” strain JA12 genome two copies of a cyclopropane fatty acyl phospholipid synthase were detected potentially allowing the synthesis and incorporation of cyclopropane fatty acyl phospholipids into the membrane. A characteristic strategy of acidophiles to further inhibit the influx of protons is the maintenance of a reversed membrane potential (inside positive membrane potential) by increasing the influx of K^+^-ions [93, 94, 99]. The genome of “*Ferrovum*” strain JA12 harbours four genes predicted to encode K^+^-transporters potentially involved in establishing an inside positive membrane potential.

The discharge of excess protons by protons pumps, antiporters or symporters is described to be another important strategy of acidophiles to maintain pH homeostasis [93]. However, in contrast to the acidophiles *A*. *ferrooxidans* ATCC 23270 [41], *Leptospirillum* group II [29] or *Picrophilus torridus* DSM 9790 [100] “*Ferrovum*” strain JA12 appears to lack genes encoding Na^+^/H^+^-antiporters. Nevertheless, like the other acidophiles [93] “*Ferrovum*” strain JA12 presumably still uses the proton pumping activity of the respiratory chain complexes of the downhill branch to extrude protons from the cytoplasm. Furthermore, the proton influx from the ATP synthase could be compensated by the consumption of protons during the reduction of oxygen at the terminal oxidase.

Due to the lack of Na^+^/H^+^-antiporters cytoplasmic buffering presumably may be of special relevance for “*Ferrovum*” strain JA12 to cope with excess protons. In this context amino acid decarboxylases for arginine, aspartate and phosphatidylserine as well as a spermidine synthase were identified. Amino acid decarboxylases and the spermidine synthase are involved in the generation of polyamines which are known to provide a cytoplasmic buffering capacity [93, 94]. The physiological relevance of the spermidine synthase has recently been shown for *A*. *caldus* in response to suboptimal pH values [95]. Furthermore, “*Ferrovum*” strain JA12 may also benefit from the buffering capacity of polyphosphates [94, 101, 102] or of the products of the urea hydrolysis as described for the gastric pathogens *Helicobacter pylori* [103] and *Yersinia enterolytica* [104]. While ammonia may buffer one proton in the cytoplasm by forming ammonium, the bicarbonate could be directed to the carbon fixation in the carboxysome of “*Ferrovum*” strain JA12 (Fig 2). Among iron oxidising bacteria this is a so far unique strategy for the adaption to acidic environments.

The genome of “*Ferrovum*” strain JA12 was also predicted to harbour a wide repertoire of chaperones and DNA repair systems (see also section Oxidative stress) presumably involved in the repair of proteins and DNA damaged due to disturbances of the intracellular pH homeostasis [93, 94, 105].

#### Strategies to cope with high metal and metalloid loads

AMD waters are usually also characterised by high concentrations of metal and metalloid ions which are particularly well liberated from ores at low pH. The inflow of the pilot plant Tzschelln was reported to contain, apart from iron, also other heavy metal ions (manganese 6.3 mg/l, nickel 0.081 mg/l, zinc 0.18 mg/l) and arsenite (0.031 mg/l) [35]. We hypothesise a potential arsenate detoxification mechanism in “*Ferrovum*” strain JA12 that appears to be relatively similar to that described for *A*. *ferrooxidans* [106, 107] and other bacteria [108] (Fig 5B and S3F Table). Apparently, “*Ferrovum*” strain JA12 is able to use an arsenate reductase to reduce arsenate to arsenite which may then be exported by an arsenite transporter. The genes predicted to encode the arsenate reductase (ArsC) and the arsenic resistance protein ArsH are located in a small gene cluster while the genes predicted to encode a putative arsenite transporter and a transcriptional regulator of the ArsR family are organised in a second cluster.

The genome of “*Ferrovum*” strain JA12 also harbours a predicted mercuric reductase (FERRO_17870) catalysing the reduction of Hg(II) to the volatile Hg^0^, but apparently no other genes of the *mer*-operon or a homologous protein to the transcriptional regulator MerR [109, 110]. Furthermore, we identified a putative copper exporting ATPase CopA (FERRO_10410) in the genome of “*Ferrovum*” strain JA12.

Apart from these specific tolerance mechanisms eleven putative cation/multidrug or heavy metal efflux pumps of the RND family [111] were detected (S3F Table). Five of them seem to be involved in the formation of a protein complex consisting of the RND family pump, a membrane fusion protein (MFP) [112] and an outer membrane factor (OMF) subunit [113]. Heavy metals could then be exported across the membranes in a single energy consuming step through a channel formed by the MFP that spans across the inner and the outer membrane formed by the MFP (Fig 5B).

In addition to these active tolerance strategies “*Ferrovum*” strain JA12 may also benefit from passive metal tolerance mechanisms that are associated with life in acidic mining environments [114]. Among these are the decreased concentration of free metal ions due to the complexation by sulfate ions [114], the reversed membrane potential of acidophiles [93, 94] (see also section Acidic environment) and the competition of the surface binding sites between metal ions and protons at low pH [115].

Overall, the metal concentrations of the pilot plant appeared to be low in comparison to other habitats [116]. However, it should be noted that other “*Ferrovum*” strains have also been detected in habitats with higher concentrations of dissolved heavy metal ions such as copper (35 mg/l) and zinc ions (50 mg/l) [9], cadmium (0.3 mg/l, [14]), cobalt (0.37 mg/l, [13]) and arsenic (2.2 mg/l, [11]).

#### Oxidative stress

Acidophilic iron oxidisers are in constant danger of oxidative stress and damages caused by reactive oxygen species (ROS) due to the high concentration of redox-active metals like iron in their natural environments and, at the same time, their need of high oxidation rates in order to maintain the cellular metabolism [117, 118]. ROS such as hydrogen peroxide, the superoxide radical or the hydroxyl radical cause either direct oxidative damage to DNA, proteins and membranes or indirect damage *via* the formation of organic peroxides from alcoholic groups. The genome analysis indicates that “*Ferrovum*” strain JA12 is equipped with a wide repertoire of genes putatively involved in the detoxification of ROS and the repair of damaged biomolecules (Fig 5C and S3F Table).

“*Ferrovum*” strain JA12 appears to convert superoxide radicals into hydrogen peroxide using the predicted superoxide dismutase of the manganese/iron type. The resulting hydrogen peroxide may then be detoxified *via* a DNA-binding ferritin-like protein or *via* one of the two thiol peroxidases (atypical 2-Cys-peroxiredoxin, 1-Cys-peroxiredoxin) similar to the acidophiles *Sulfolobus solfataricus* [119], *Acidithiomicrobium* spp., *Alicyclobacillus* spp. and *Sulfobacillus* spp. [120]. The peroxiredoxins are described as typical markers for oxidative stress which oxidise hydroperoxides *via* conserved cysteine residues [121].

In case of oxidative damage to its DNA “*Ferrovum*” strain JA12 was predicted to use repair systems similar to those described for other acidophiles including the base excision repair, nucleotide excision repair, mismatch repair and the classical RecA/LexA-dependent SOS response.

The base excision repair was predicted to involve a DNA-3-methyladenine glycosylase, an A/G-specific DNA-adenine glycosylase and a predicted endonuclease III in “*Ferrovum*” strain JA12. The nucleotide excision repair appears to be achieved *via* the classic excinuclease UvrABC and may further be complemented by a transcription-repair coupling factor, an ATP-dependent DNA helicase and a nudix hydrolase family enzyme. The latter has been described to be involved in the degradation of oxidised, potentially mutagenic nucleotides [122]. With respect to the DNA mismatch repair “*Ferrovum*” strain JA12 apparently supplements the conserved minimal complex consisting of MutL and MutS by a T/G mismatch-specific endonuclease.

Apart from DNA, the sulfur containing residues cysteine and methionine in proteins are sensitive to oxidative damage. The genome of “*Ferrovum*” strain JA12 was predicted to harbour several repair systems to restore the original redox state of cysteine residues in proteins. The classical thioredoxin/thioredoxin reductase system appears to be represented by multiple copies of thioredoxin encoding genes and a thioredoxin reductase encoding gene. The thiol:disulfide interchange proteins DsbA and DsbD were predicted to be involved in oxidative protein folding in the periplasm as described in other bacteria [123] and they have also been discussed to be part of the oxidative stress response in the acidophiles *Acidithiobacillus* spp., *A*. *cryptum*, and *Leptospirillum* spp. [120]. In contrast to many other acidophiles [120] the “*Ferrovum*” strain JA12 genome appeared neither to encode the methionine-S-sulfoxide reductase MsrA nor the methionine-R-sulfoxide reductase MsrB to cope with oxidatively damaged methionine.

The removal of organic peroxides was predicted to be mediated by peroxiredoxins (AhpC). While five of the six *ahpC*-gene copies were distributed in the genome, one copy (FERRO_08490) was found to be co-located with the gene predicted to encode the alkyl hydroperoxide reductase (AhpF, FERRO_08500) which is likely involved in restoring the active state of the peroxiredoxins. Furthermore, “*Ferrovum*” strain JA12 may use glutathione-dependent systems to cope with damaged proteins and lipids, such as the glutathione peroxidase. Genes involved in the glutathione biosynthesis (glutamate-cysteine ligase, the glutathione synthase, γ-glutamyltransferase) were identified, but it remains unclear how “*Ferrovum*” strain JA12 regenerates glutathione from glutathione disulfide since its genome apparently lacks a gene encoding the NAD(P)H-dependent glutathione reductase.

### Horizontal gene transfer

A range of mobile genetic elements was predicted in the genome of “*Ferrovum*” strain JA12 including 22 transposases and eight integrases representing at least ten different insertion element classes, twelve phage-related proteins, three proteins putatively involved in plasmid stabilisation and inheritance, and the predicted mobile mystery proteins A and B (TIGR02612, TIGR02613) (S3G Table).

Furthermore, genes predicted to encode homologous proteins to the VirB/D4 secretion system were detected at three different loci in the genome of “*Ferrovum*” strain JA12 (S5 Fig). The VirB/D4 secretion system is a subclass of the type IV secretion system named after the type IV secretion system in *Agrobacterium tumefaciens* [124]. Locus 1 in the genome of “*Ferrovum*” strain JA12 was predicted to encode ten of the twelve VirB/D4 proteins of *A*. *tumefaciens* which were found to be arranged in a slightly different order in comparison to *A*. *tumefaciens*. However, the genes encoding a lytic transglycosylase (VirB1) and the lipoprotein VirB7 were not identified in the cluster of locus 1. The second locus was also predicted to harbour the nearly complete set of VirB/D4 proteins, again missing the genes encoding VirB1 and B7, while the third locus only contained the genes encoding VirB5 and B6. According to studies in *A*. *tumefaciens* all 12 proteins are necessary to form the functional secretion system [125, 126]. Hence, it remains unclear whether “*Ferrovum*” strain JA12 may harbour a functional type IV secretion system and a functional conjugational machinery. However, the co-localised integrase in locus 1 may indicate that the VirB/D4 encoding genes in “*Ferrovum*” strain JA12 are part of an integrative and conjugative element (ICE) which has been reported to often contain the genes of the type IV secretions system [127].

The presence of both predicted mobile genetic elements and genes putatively associated with conjugational DNA-transfer in an otherwise small genome may indicate that horizontal gene transfer (HGT) *via* transduction and conjugation may have played an important role in the genome evolution and for the acquisition of metabolic traits in “*Ferrovum*” strain JA12.

## Conclusions

The genomic approach successfully extended our current knowledge of the physiological capacity of the genus “*Ferrovum*” by providing a comprehensive description of the metabolic potential of the novel “*Ferrovum*” strain JA12. Apparently, “*Ferrovum*” strain JA12 is able to maintain a chemolithoautotrophic lifestyle by utilising available carbon, nitrogen, sulfur, phosphate and energy sources in the pilot plant Tzschelln. The absence of proton symporters and antiporters may explain why “*Ferrovum*” has been observed to prefer higher pH values than other acidophiles such as *Acidithiobacillus* spp. and *Leptospirillum* spp. The distinguishing genome and metabolic features between the type strain “*F*. *myxofaciens*” P3G and “*Ferrovum*” strain JA12 indicate a metabolic diversity within the genus “*Ferrovum*” that may be the fundament for the widespread distribution of these acidophiles. Furthermore, the identification of diverse mobile genetic elements and the reduced genome size of “*Ferrovum*” strain JA12 revealed potential driving forces of the genome evolution and speciation in “*Ferrovum*”. The present study will be used as basis for a future comparative genome study which will address the latter two aspects.

## Methods

### Origin and cultivation of “*Ferrovum*” strain JA12

The iron oxidising mixed culture JA12 was obtained during a previous study by plating a water sample derived from the mine water treatment plant Tzschelln in Lusatia (Saxony, Germany) on overlay plates based on the artificial pilot water medium (APPW) [33] (Permission for sampling the pilot plant water for microbiological purposes was granted by G.E.O.S. Ingenieurgesellschaft mbH, Gewerbepark Schwarze Kiefern, Halsbrücke, Germany). Chemical composition of the pilot plant inflow has been reported previously [35]. Culture JA12 was cultivated in artificial pilot plant water medium and periodically transferred into fresh medium [33] with ferrous iron sulfate as electron donor. Terminal restriction fragment length polymorphism (TRFLP) and sequencing of 16S rRNA gene fragments revealed that culture JA12 consisted of the iron oxidiser “*Ferrovum*” strain JA12 and the heterotroph *Acidiphilium* strain JA12-A1 [33]. Ferrous iron oxidation was therefore used as indicator for cellular growth of the iron oxidiser “*Ferrovum*” strain JA12 with quantification of ferrous iron being achieved *via* the ferrozine method [128].

Cells were harvested by centrifugation (5,000 x *g*) and washed with 50 mM oxalic acid in 0.9% (w/v) NaCl. Genomic DNA was isolated either with the UltraClean^®^ microbial DNA isolation kit (MOBIO Laboratories Inc.) for next generation sequencing or with the MasterPure^™^ Gram Positive DNA Purification Kit (Epicentre Technologies Corp., WI, USA) to obtain template DNA for PCRs to achieve gap closure.

During the course of a follow-on study, and following the genome analysis reported here, “*Ferrovum*” strain JA12 was lost in culture JA12 (“*Ferrovum*” strain JA12 was no longer detectable by a specific PCR amplification of a 16S rRNA gene fragment of “*Ferrovum*” strain JA12).

### Genome sequencing, assembly and annotation

In order to assemble the genome of the iron oxidiser “*Ferrovum*” strain JA12 from the metagenome reads of the mixed culture JA12 the contaminating *Acidiphilium* strain JA12-A1 was brought into pure culture and its genome was sequenced [34]. Genome sequencing of “*Ferrovum*” strain JA12 was performed at the Göttingen Genomics Laboratory (G2L, Göttingen University, Germany) *via* a hybrid approach using the 454 GS-FLX Titanium XL system (Titanium GS70 chemistry, Roche Life Science) and the Genome Analyzer II (Illumina). The shotgun libraries were prepared according to the manufacturers’ protocols. This involved, in the case of the Illumina platform, the use of the Nextera XT library preparation kit and resulted in a mean insert size of 140.15 bp. The 3,113,232 112 bp paired-end Illumina sequence reads were pre-processed using Trimmomatic with quality filter Phred33 [129] resulting in trimmed sequence reads with a mean length of 92.34 bp. Sequence reads that mapped to the genome of *Acidiphilium* strain JA12-A1 were removed from the dataset. A draft genome of “*Ferrovum*” strain JA12 was assembled *de novo* based on 61,459 454 shotgun reads (genome coverage: 12 x) and 2,089,798 trimmed paired-end Illumina reads (genome coverage: 115 x) using the Newbler 2.6 (Roche Life Science) and the MIRA 3.4 [130] software. The raw data were manually inspected and quality checked using the Staden Package GAP4 [131], FastQC version 0.10 [132] and Qualimap [133]. Specific PCR primers for gap closure were designed based on the GAP 4 inspection. Specificity of the designed primers was checked by blastn search against the genome sequence. Assembly and subsequent gap closing procedure resulted in a nearly complete genome sequence of “*Ferrovum*” strain JA12 consisting of three contigs.

Automatic annotation was conducted at Göttingen Genomics Laboratory using PRODIGAL [134] predicting coding sequences. tRNA genes were predicted using tRNAscan-SE [135] adjusted to bacterial genes and ARAGORN [136]. rRNA genes were predicted using RNAmmer [137]. The predicted coding sequences were annotated using in-house scripts at the Göttingen Genomics Laboratory. Blastn searches were conducted against the downloaded databases of Swiss-Prot, TrEMBL [138] and InterPro [139] using a cut-off value of 1e^-20^ and subsequent filtering for the best hit. Further automatic annotation was performed within the pipeline of the integrated microbial genomes-expert review (IMG/ER) system [140, 141].

Transmembrane helices in protein sequences were identified by TMHMM 2.0 using the default settings ([142], http://www.cbs.dtu.dk/services/TMHMM/; accessed 8 January 2015). Regulatory sequences within the urease gene cluster were predicted using the software FGENESB with default settings (Softberry Inc., Mount Kisco, NY, USA). Metabolic pathways in “*Ferrovum*” strain JA12 were inferred using the KEGG database [143, 144] and the NCBI conserved domain search [145, 146]. The predicted metabolic traits were subsequently compared to other acidophilic and neutrophilic iron oxidisers and to individual non-iron oxidisers. Accession numbers of genomes used in this context are listed in S4 Table.

### Visualisation of the nearly complete genome

The three contigs of the nearly complete genome sequence of “*Ferrovum*” strain JA12 were concatenated using the BLAST Ring Image Generator (BRIG) [47] in order to facilitate visualisation of the GC content and GC-skew. A Blastn-based genome comparison with the draft genome sequence of the type strain “*Ferrovum myxofaciens*” P3G was conducted with BRIG using the default Blast options of the program and running BLAST+ [147] version 2.2.30. Identified matches were added to the circular plot of the genome sequence of “*Ferrovum*” strain JA12.

### Phylogenetic analysis

Phylogeny was inferred based on the determination of three genome-based phylogenetic indicators (DDH, ANIb, tetra) and the calculation of a 16S rRNA gene sequences-based phylogenetic tree. See S1 Fig for a detailed description of procedure applied for the calculation of a 16S rRNA gene-based phylogenetic tree.

The DNA-DNA-Hybridisation (DDH) value was estimated *in silico* using the genome-to-genome-distance-calculator (GGDC2.0, [36, 148], http://ggdc.dsmz.de/distcalc2.php) with default settings. The DDH was inferred from the results of formula 2. The calculation of the average nucleotide identity based on BLAST version 2.2.26 (ANIb) and the regression of the tetranucleotide composition (tetra) were conducted in JSpecies [39] using default settings. BLAST version 2.2.26 was downloaded from the NCBI FTP server on 10 March 2015.

### Prediction of mobile genetic elements

Insertion elements were predicted using the ORFminer annotation pipeline (in-house pipeline, Center for Bioinformatics and Genome Biology, Fundación Ciencia y Vida, Santiago, Chile) including TnpPred [149]. The results were complemented using ISsaga ([150], www.is.biotoul.fr; accessed 1 April 2015) to further classify transposases and integrases. Candidates that were only predicted by one of the programs were investigated manually in more detail.

Furthermore, a blastp search against the ACLAME database ([151], http://aclame.ulb.ac.be/; accessed 31 March 2015) was conducted to detect virus and prophage-associated genes (Prophinder, [152]) using default settings.

### Nucleotide sequence accession number

The nearly complete genome sequence of “*Ferrovum*” strain JA12 is accessible at DDBJ/EMBL/GenBank under the accession number LJWX00000000.

## Supporting Information

S1 Fig 16S rRNA gene-based dendrogram. The dendrogram includes iron oxidising and non-iron oxidising members of the phylum *Proteobacteria* and uses the non-iron oxidising deltaproteobacterium *Geobacter metallireducens* as outgroup. 16S rRNA gene sequences were imported into the ARB software program and aligned to other proteobacterial 16S rRNA gene sequences using the automated alignment tool within ARB [153]. Calculation of phylogenetic trees based on these sequence alignments was conducted within MEGA6 [154] using the neighbor-joining method with Jukes-Cantor corrections [155], as well as the maximum likelihood and parsimony algorithms. For each of the phylogenetic analyses in this study, the grouping of strains and environmental clones within the different clusters of the tree was identical for all three phylogenetic methods for calculating trees. However, those branching points within a tree that were not supported by each of the three algorithms were collapsed within the neighbor-joining tree using a strict consensus rule until the branching was supported in all three analyses. The neighbor-joining tree was chosen for depicting the phylogenetic relationship of the 16S rRNA gene clones and strains. Numbers next to branches indicate the percentage of replicates (out of 1,000 bootstrap trees) in which the associated taxa clustered together [156]. The dendrogram underlines the closer relationship of the “*Ferrovum*” strains P3G and JA12 to the neutrophilic iron oxidising *Betaproteobacteria* including *G*. *capsiferriformans*, *S*. *lithotrophicus* and *T*. *denitrificans* while other acidophilic iron oxidisers belong to the *Acidithiobacillia* and *Gammaproteobacteria* (i.e. *T*. *prosperus*) as described previously [23, 57].
(PDF)

S2 Fig Central carbon metabolism in “*Ferrovum*” strain JA12. (A) The predicted pathways of the central carbon metabolism in “*Ferrovum*” strain JA12 involved in the production of amino acids, nucleic acids, fatty acids, and nucleotide-activated monosaccharides are shown. The carbon dioxide fixation product 3-phosphoglycerate is predicted to be directed into the central carbon metabolism. Apparently, the amino acids serine, glycine and cysteine are formed by conversion from 3-phosphoglycerate. In the glycolysis 3-phosphoglycerate is predicted to be converted to pyruvate and further to acetyl-CoA. While pyruvate could serve as precursor for the synthesis of leucine, isoleucine, valine and alanine, acetyl-CoA appears either to serve as precursor for fatty acid biosynthesis or to be directed into the citrate cycle. The intermediates of the citrate cycle oxaloacetate and α-ketoglutarate are precursors for the biosynthesis of the amino acids aspartate, asparagine, arginine, lysine, threonine, methionine, and of glutamate, glutamine, proline, respectively. The carbon fixation product 3-phosphoglycerate could be converted to glucose-6-phosphate (gluconeogenesis) which is also an intermediate of the pentose phosphate pathway. The pentose phosphate pathway intermediate erythrose-4-phosphate and pyruvate resulting from glycolytic reactions may serve as precursors for the synthesis of the aromatic amino acids phenylalanine, tyrosine and tryptophan. The conversions of glucose in the pentose phosphate pathway also lead to the production of phosphoribosyl pyrophosphate which is the general precursor for the synthesis of pyrimidines, purines and the amino acid histidine. Glucose-6-phosphate could also be converted to nucleotide-activated derivates that are predicted to serve as the precursors for the syntheses of peptidoglycan and lipopolysaccharides of the cell envelope and potentially also for the synthesis of exopolysaccharides of the EPS. The genes predicted to be involved in the pathways are listed in S3 Table. (B) The gene cluster (FERRO_01000—FERRO_01150) thought to be involved in the formation of nucleotide-activated monosaccharides for the synthesis of exopolysaccharides encodes hypothetical proteins (hp, black), glycosyltransferases (gt, orange), enzymes of the nucleotide sugar metabolism that are involved in the formation of the putative precursors (green) and other enzymes of the nucleotide sugar metabolism without specified function (light blue). Apparently, the formation of the precursors GDP-mannose (GDP-Man), dTDP-rhamnose (dTDP-Rha), UDP-glucose (UDP-Glc), UDP-galactose (UDP-Gal), UDP-glucuronic acid (UDP-GlcA) and UDP-galacturonic acid (UDP-GalA) also involves enzymes that are not encoded by the gene cluster (white, see also S3A Table). The precursors are predicted to be transferred to the growing polysaccharide chain by glycosyltransferases though it remains unclear whether the glycosyltransferases encoded in the gene cluster could be involved in this processes.
(PDF)

S3 Fig Predicted transmembrane regions in cytochromes in “*Ferrovum*” strain JA12 and *A*. *ferrooxidans* ATCC 23270. Transmembrane regions were predicted in cytochromes potentially involved in the ferrous iron oxidation in “*Ferrovum*” strain JA12 and in Cyc2, Cyc1 and CycA-1 of *A*. *ferrooxidans* ATCC 23270 using TMHMM 2.0. The plots show the probability of the amino acid residues of the cytochromes to belong to transmembrane helices. The inferred location of the residues (transmembrane, inside, outside) is indicated by the colours red, blue and purple, respectively. (A) In the Cyc2-like high molecular mass cytochrome (FERRO_13930) of “*Ferrovum*” strain JA12 a transmembrane helix was predicted in the N-terminal region similar to Cyc2 of *A*. *ferrooxidans* (AFE_3153) indicating that both cytochromes are membrane bound. (B) In contrast to Cyc1 no transmembrane helices were predicted for *c*-type cytochromes (FERRO_02680, FERRO_02750) in “*Ferrovum*” strain JA12 indicating that they are all soluble cytochromes like CycA-1.
(PDF)

S4 Fig Inferring the role of cytochromes of “*Ferrovum*” strain JA12 for the ferrous iron oxidation. (A) A dendrogram of high potential iron-sulfur proteins from *Acidithiobacillus* spp. and “*Ferrovum*” strain JA12 was calculated by aligning the protein sequences using ClustalW (75 positions). The phylogeny was inferred by the Maximum Likelihood method based on the Whelan and Goldman model [157] using the MEGA6 [154] (bootstrap: 1000 replicates). In the genome of “*Ferrovum*” strain JA12 a hypothetical protein (FERRO_02670) was identified that contained the conserved cysteine residues of high potential iron-sulfur proteins [158]. The predicted high potential iron-sulfur protein (FERRO_02670) shared a sequence identity of 32% to the high potential iron-sulfur protein Hip of *A*. *ferrooxidans* ATCC 23270 (AFE_2732) and of 28% to the iron oxidase Iro of *A*. *ferrivorans* SS3 (Acife_3266). In order to infer a potential physiological role of the predicted high potential iron-sulfur protein in “*Ferrovum*” strain JA12 a dendrogram was calculated based on the protein sequences of high potential iron-sulfur proteins from *Acidithiobacillus* spp. The branches containing the iron oxidase Iro (Acife_3266) or Hip (AFE_2732) are indicated. The predicted high potential iron-sulfur protein of “*Ferrovum*” strain JA12 (FERRO_02670) represents the outgroup to the high potential iron-sulfur proteins from *Acidithiobacillus* spp. Hence, it remains unclear whether the predicted high potential iron-sulfur protein may be involved in the iron oxidation of “*Ferrovum*” strain JA12. (B) In order to elucidate the participation of the soluble *c*-type cytochromes of “*Ferrovum*” strain JA12 (FERRO_02680, FERRO_02750) either in the downhill electron transfer or the uphill electron transfer a dendrogram was calculated including the protein sequences of Cyc1 and CycA-1 of *Acidithiobacillus* spp. and homologous cytochromes of *Thiobacillus prosperus* V6. The protein sequences were aligned using ClustalW (175 positions). The phylogeny was inferred by the Maximum Likelihood method based on the Whelan and Goldman model [157] using the MEGA6 [154] (bootstrap: 1000 replicates). The *c*-type cytochrome encoded by FERRO_02680 forms an outgroup to all other cytochromes. It shares a sequence identity of 35% to CycA-1 (AFE_3107) and of 32% to Cyc1 (AFE_3152). The other *c*-type cytochrome of strain JA12 (FERRO_02750) forms a subcluster with homologous proteins of the acidophilic iron oxidiser *Thiobacillus prosperus* V6 and shares 40% identical positions with CycA-1 and 30% with Cyc1. Its apparently higher similarity to CycA-1 may indicate its potential involvement in the uphill electron transfer like CycA-1, though it remains only hypothetical. (C) “*Ferrovum*” strain JA12 is predicted to use the *cbb*_3_-type cytochrome *c* oxidase as terminal oxidase in the ferrous iron oxidation. The gene cluster encoding the predicted to subunit I (FERRO_02570, orange) and subunit II (FERRO_02560, green) of the *cbb*_3_-type cytochrome *c* oxidase is shown. Co-localised *c*-type cytochromes (blue) could substitute the missing subunit III in the *cbb*_3_-type cytochrome *c* oxidase.
(PDF)

S5 Fig VirB/D4 type IV secretion system in “*Ferrovum*” strain JA12. The loci in the “*Ferrovum*” strain JA12 genome encoding proteins of the VirB/D4 type IV secretion system were compared with the locus in *Agrobacterium tumefaciens*. (A) The *virB*-operon in *A*. *tumefaciens* is based on [124]. (B) The *trb*-genes in strain JA12 are homologous to the *vir*-genes in *A*. *tumefaciens* as indicated in parentheses. Genes coloured in orange, grey, green or black are predicted to encode proteins of the VirB/D4 type IV secretion system, proteins presumably not related to the VirB/D4 system, mobile genetic elements (integrase, transposase) or hypothetical proteins, respectively.
(PDF)

S1 Table Protein-coding genes of “*Ferrovum*” strain JA12 in COG categories. The protein-coding genes were assigned to the COG classes *via* the IMG/ER [141] prediction pipeline (17 February 2014). The percentage is based on the total number of protein-coding genes assigned to the COG categories (1,462).
(DOCX)

S2 Table General genome features of selected iron oxidising prokaryotes. Information on the genome features of selected iron oxidising prokaryotes representing various taxa according to the Integrated Microbial Genomes (IMG) database from January, 22, 2015.
(XLSX)

S3 Table Gene lists by predicted metabolic role in “*Ferrovum*” strain JA12. (A) Carbon metabolism: carbon fixation, central carbon metabolism, uptake systems for organic carbon compounds, biosynthesis of cell envelope polysaccharides, storage of organic carbon compounds, biosynthesis of fatty acids, biosynthesis of amino acids, purine and pyrimidine metabolism. (B) Nitrogen metabolism: nitrogen assimilation, uptake systems for nitrogen compounds. (C) Phosphate metabolism: phosphate uptake, polyphosphate storage. (D) Sulfur metabolism: sulfate uptake and assimilation. (E) Energy metabolism: ferrous iron oxidation, other redox reactions connected to the quinol pool, putative formate dehydrogenase. (F) Stress management: acid stress, heavy metals and metalloids, oxidative stress. (G) Predicted mobile genetic elements: predicted insertion elements, putative phage-associated proteins, plasmid stabilisation and inheritance, other putative mobile genetic elements, VirB/D4 type IV secretion pathway. The predictions were performed using ISsaga [150], TnpPred [149] and Profinder [151, 152].
(XLSX)

S4 Table List of nucleotide accession numbers of genomes mentioned in the study. (DOCX)
