# Supplementary figures and images for: Genome Analysis of the Biotechnologically Relevant Acidophilic Iron Oxidising Strain JA12 Indicates Phylogenetic and Metabolic Diversity within the Novel Genus “Ferrovum”
Source: PLoS One. 2016 Jan 25;11(1):e0146832. doi: 10.1371/journal.pone.0146832 (PMC4725956; doi:10.1371/journal.pone.0146832)

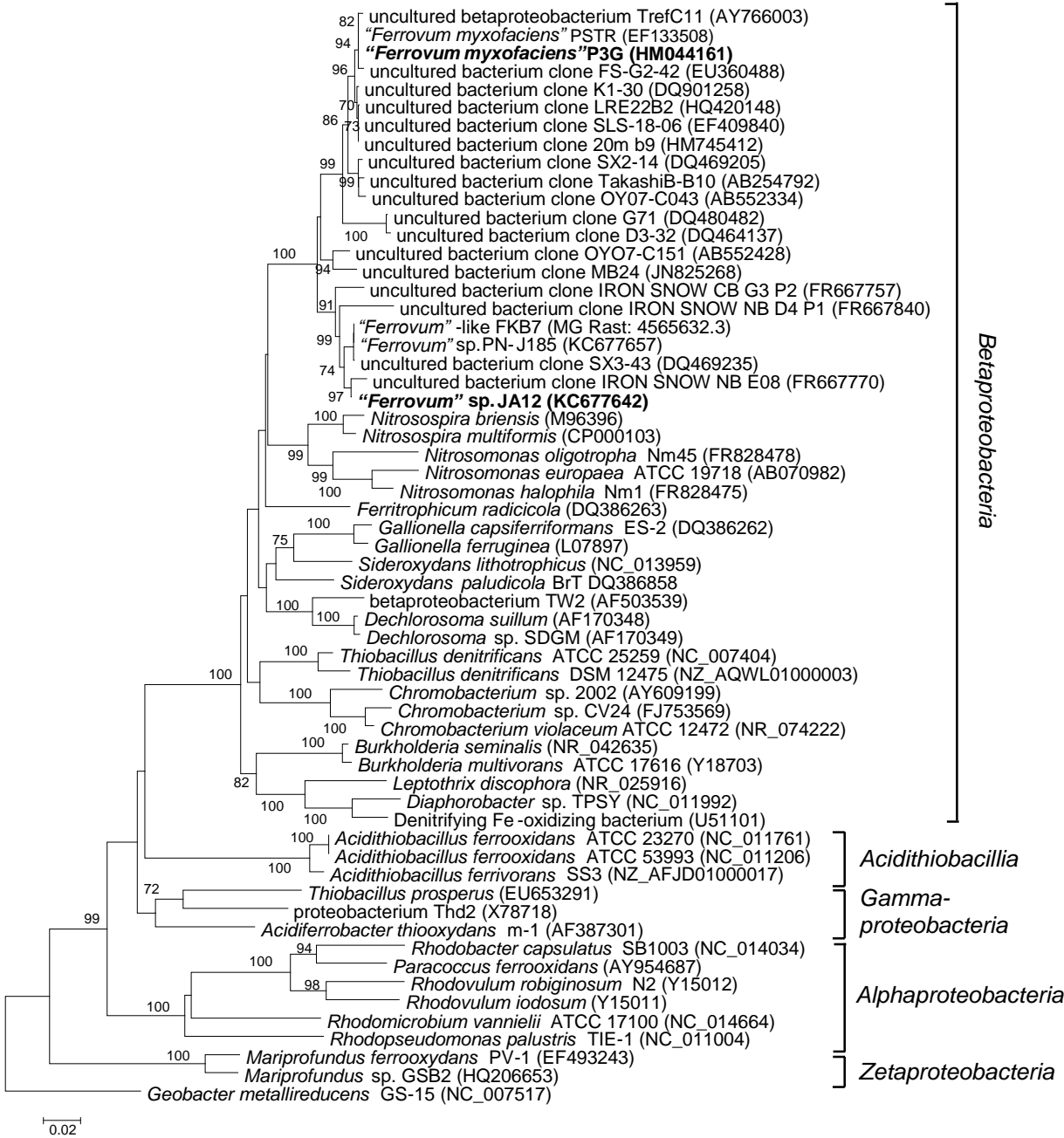

Supplement: S1 Fig — The dendrogram includes iron oxidising and non-iron oxidising members of the phylum Proteobacteria and uses the non-iron oxidising deltaproteobacterium Geobacter metallireducens as outgroup. 16S rRNA gene sequences were imported into the ARB software program and aligned to other proteobacterial 16S rRNA gene sequences using the automated alignment tool within ARB [153]. Calculation of phylogenetic trees based on these sequence alignments was conducted within MEGA6 [154] using the neighbor-joining method with Jukes-Cantor corrections [155], as well as the maximum likelihood and parsimony algorithms. For each of the phylogenetic analyses in this study, the grouping of strains and environmental clones within the different clusters of the tree was identical for all three phylogenetic methods for calculating trees. However, those branching points within a tree that were not supported by each of the three algorithms were collapsed within the neighbor-joining tree using a strict consensus rule until the branching was supported in all three analyses. The neighbor-joining tree was chosen for depicting the phylogenetic relationship of the 16S rRNA gene clones and strains. Numbers next to branches indicate the percentage of replicates (out of 1,000 bootstrap trees) in which the associated taxa clustered together [156]. The dendrogram underlines the closer relationship of the “Ferrovum” strains P3G and JA12 to the neutrophilic iron oxidising Betaproteobacteria including G. capsiferriformans, S. lithotrophicus and T. denitrificans while other acidophilic iron oxidisers belong to the Acidithiobacillia and Gammaproteobacteria (i.e. T. prosperus) as described previously [23, 57]. (PDF) [file pone.0146832.s001.pdf]

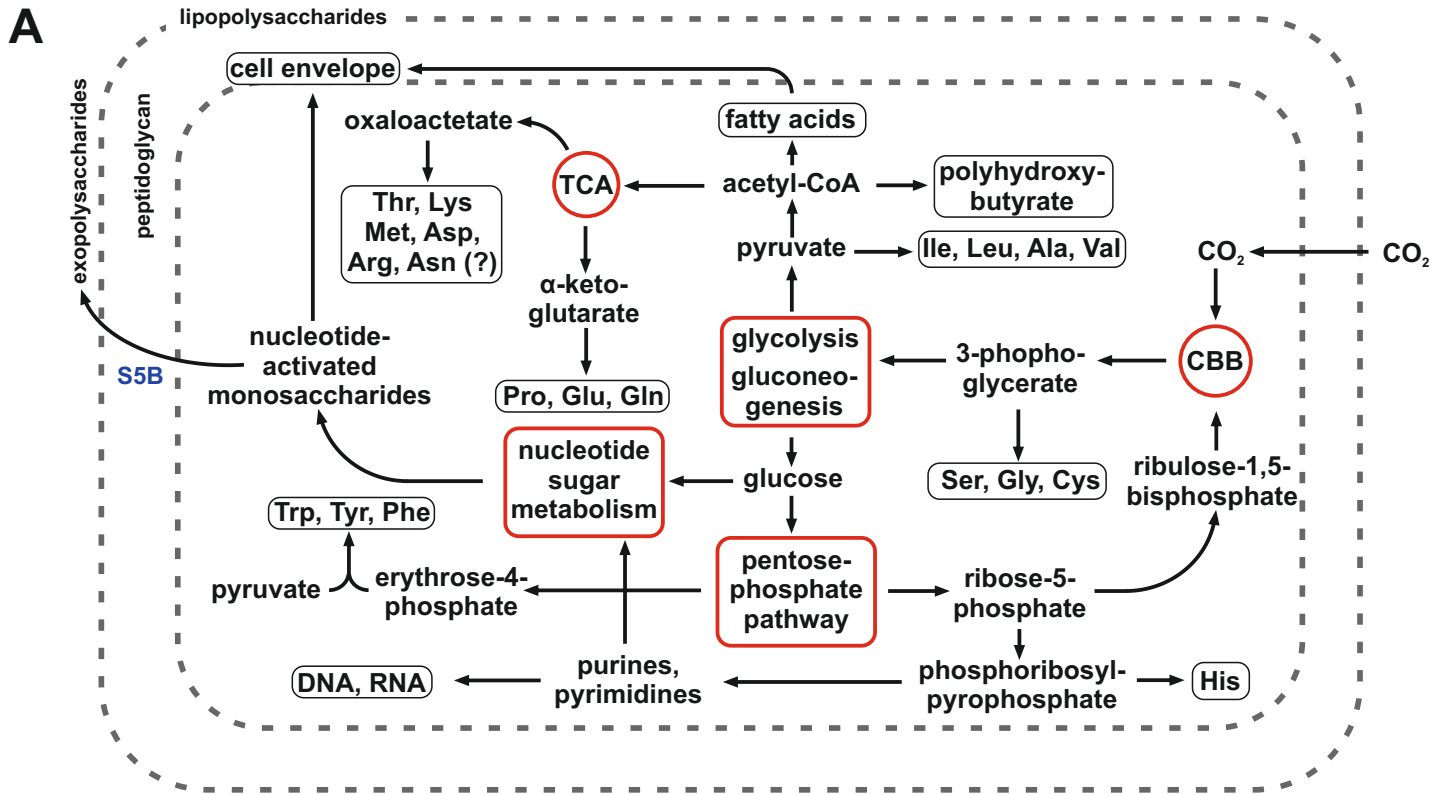

**B** FERRO\_01000 - FERRO\_01150

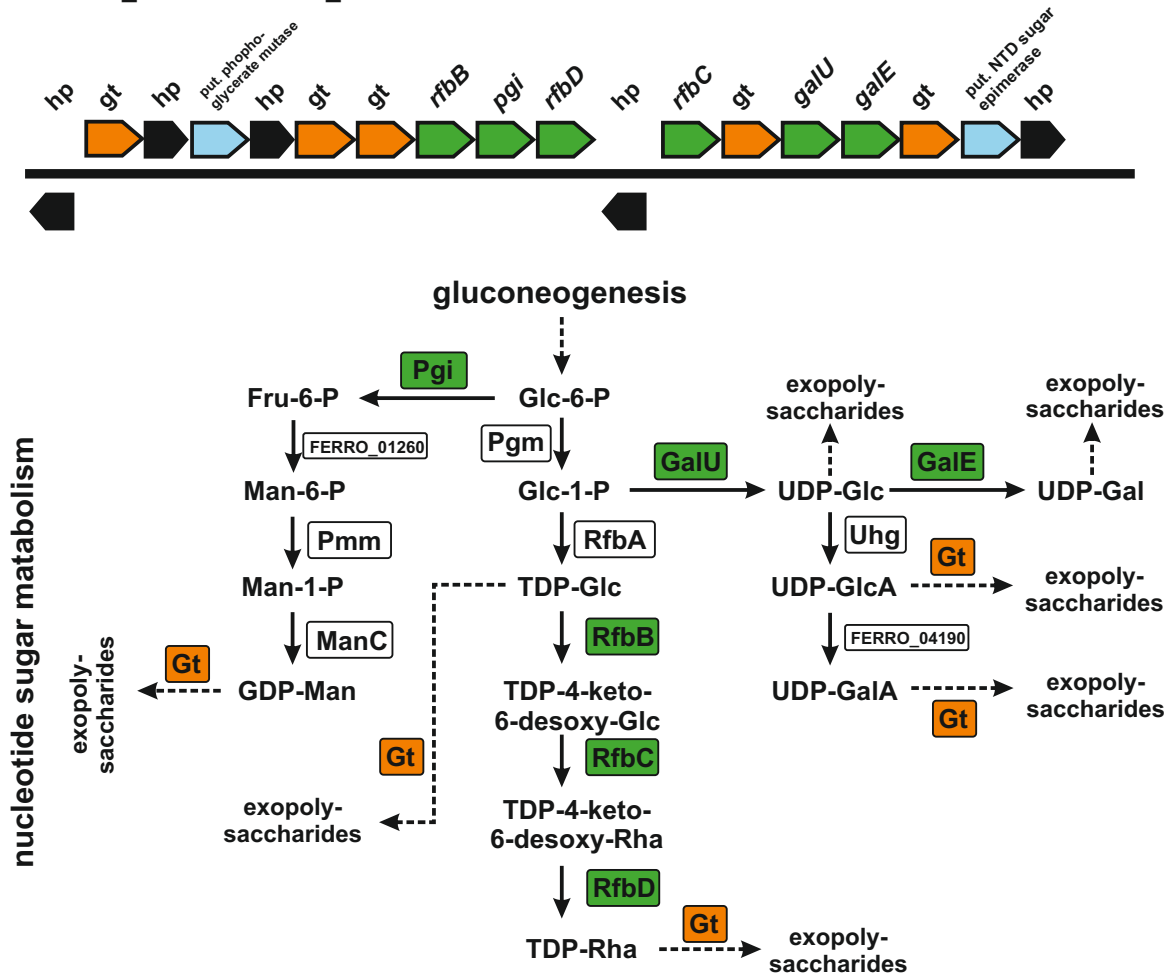

Supplement: S2 Fig — (A) The predicted pathways of the central carbon metabolism in “Ferrovum” strain JA12 involved in the production of amino acids, nucleic acids, fatty acids, and nucleotide-activated monosaccharides are shown. The carbon dioxide fixation product 3-phosphoglycerate is predicted to be directed into the central carbon metabolism. Apparently, the amino acids serine, glycine and cysteine are formed by conversion from 3-phosphoglycerate. In the glycolysis 3-phosphoglycerate is predicted to be converted to pyruvate and further to acetyl-CoA. While pyruvate could serve as precursor for the synthesis of leucine, isoleucine, valine and alanine, acetyl-CoA appears either to serve as precursor for fatty acid biosynthesis or to be directed into the citrate cycle. The intermediates of the citrate cycle oxaloacetate and α-ketoglutarate are precursors for the biosynthesis of the amino acids aspartate, asparagine, arginine, lysine, threonine, methionine, and of glutamate, glutamine, proline, respectively. The carbon fixation product 3-phosphoglycerate could be converted to glucose-6-phosphate (gluconeogenesis) which is also an intermediate of the pentose phosphate pathway. The pentose phosphate pathway intermediate erythrose-4-phosphate and pyruvate resulting from glycolytic reactions may serve as precursors for the synthesis of the aromatic amino acids phenylalanine, tyrosine and tryptophan. The conversions of glucose in the pentose phosphate pathway also lead to the production of phosphoribosyl pyrophosphate which is the general precursor for the synthesis of pyrimidines, purines and the amino acid histidine. Glucose-6-phosphate could also be converted to nucleotide-activated derivates that are predicted to serve as the precursors for the syntheses of peptidoglycan and lipopolysaccharides of the cell envelope and potentially also for the synthesis of exopolysaccharides of the EPS. The genes predicted to be involved in the pathways are listed in S3 Table. (B) The gene cluster (FERRO [file pone.0146832.s002.pdf]

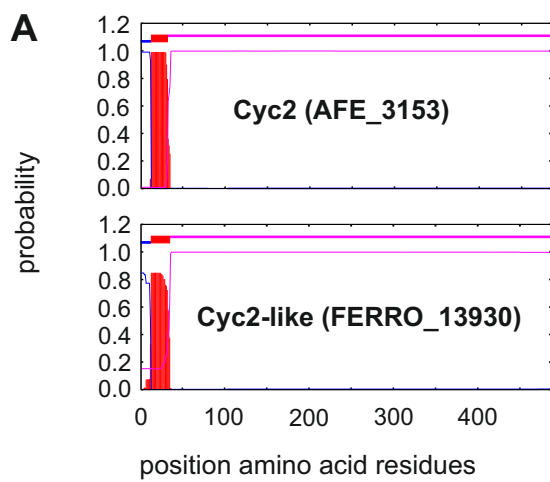

predicted location of amino acid residues:

transmembrane — red  
inside — blue  
outside — magenta

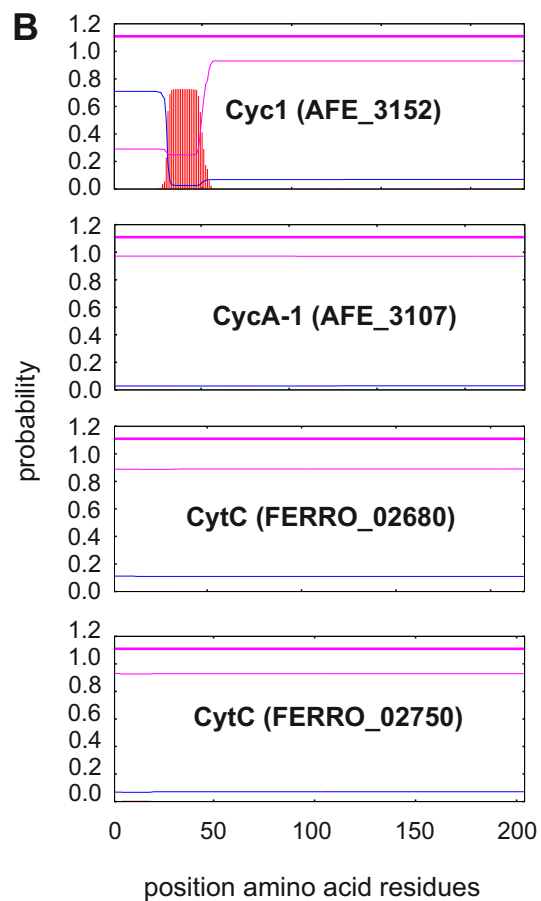

Supplement: S3 Fig — Transmembrane regions were predicted in cytochromes potentially involved in the ferrous iron oxidation in “Ferrovum” strain JA12 and in Cyc2, Cyc1 and CycA-1 of A. ferrooxidans ATCC 23270 using TMHMM 2.0. The plots show the probability of the amino acid residues of the cytochromes to belong to transmembrane helices. The inferred location of the residues (transmembrane, inside, outside) is indicated by the colours red, blue and purple, respectively. (A) In the Cyc2-like high molecular mass cytochrome (FERRO_13930) of “Ferrovum” strain JA12 a transmembrane helix was predicted in the N-terminal region similar to Cyc2 of A. ferrooxidans (AFE_3153) indicating that both cytochromes are membrane bound. (B) In contrast to Cyc1 no transmembrane helices were predicted for c-type cytochromes (FERRO_02680, FERRO_02750) in “Ferrovum” strain JA12 indicating that they are all soluble cytochromes like CycA-1. (PDF) [file pone.0146832.s003.pdf]

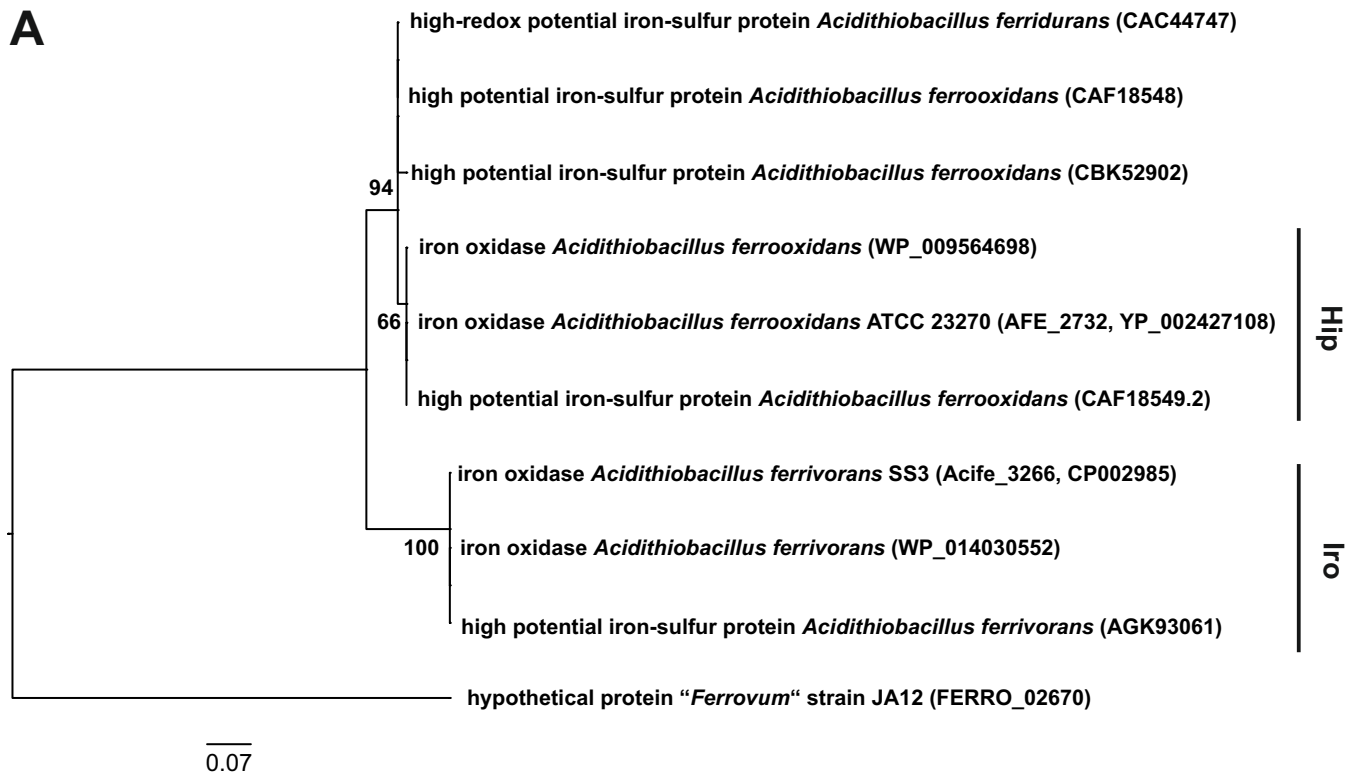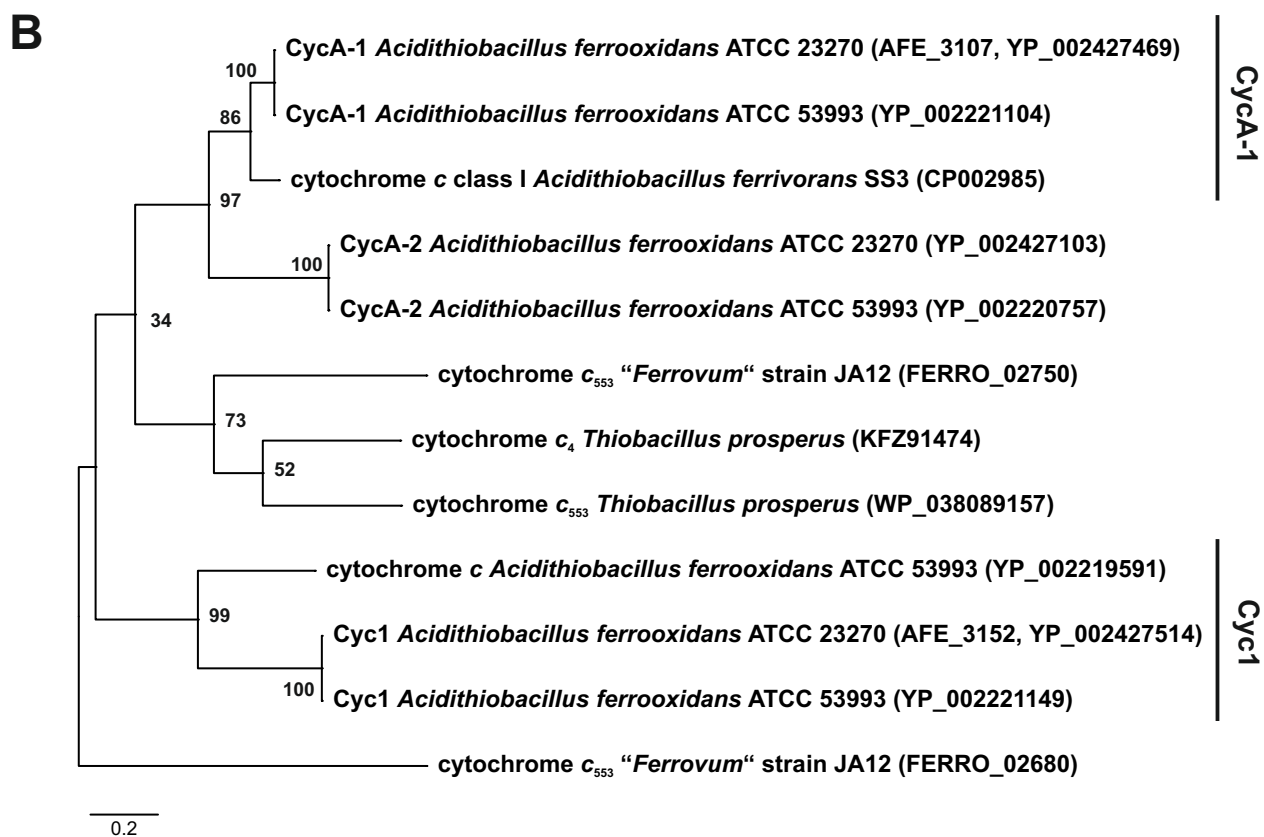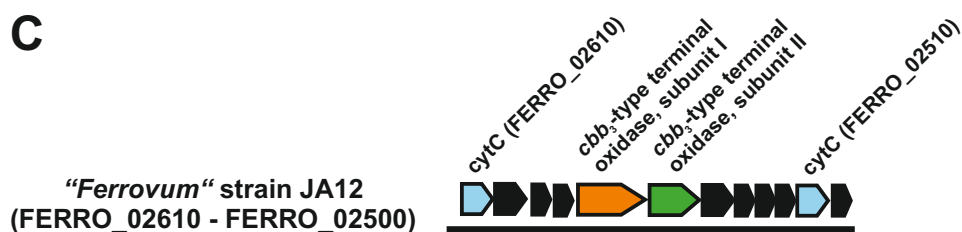

Supplement: S4 Fig — (A) A dendrogram of high potential iron-sulfur proteins from Acidithiobacillus spp. and “Ferrovum” strain JA12 was calculated by aligning the protein sequences using ClustalW (75 positions). The phylogeny was inferred by the Maximum Likelihood method based on the Whelan and Goldman model [157] using the MEGA6 [154] (bootstrap: 1000 replicates). In the genome of “Ferrovum” strain JA12 a hypothetical protein (FERRO_02670) was identified that contained the conserved cysteine residues of high potential iron-sulfur proteins [158]. The predicted high potential iron-sulfur protein (FERRO_02670) shared a sequence identity of 32% to the high potential iron-sulfur protein Hip of A. ferrooxidans ATCC 23270 (AFE_2732) and of 28% to the iron oxidase Iro of A. ferrivorans SS3 (Acife_3266). In order to infer a potential physiological role of the predicted high potential iron-sulfur protein in “Ferrovum” strain JA12 a dendrogram was calculated based on the protein sequences of high potential iron-sulfur proteins from Acidithiobacillus spp. The branches containing the iron oxidase Iro (Acife_3266) or Hip (AFE_2732) are indicated. The predicted high potential iron-sulfur protein of “Ferrovum” strain JA12 (FERRO_02670) represents the outgroup to the high potential iron-sulfur proteins from Acidithiobacillus spp. Hence, it remains unclear whether the predicted high potential iron-sulfur protein may be involved in the iron oxidation of “Ferrovum” strain JA12. (B) In order to elucidate the participation of the soluble c-type cytochromes of “Ferrovum” strain JA12 (FERRO_02680, FERRO_02750) either in the downhill electron transfer or the uphill electron transfer a dendrogram was calculated including the protein sequences of Cyc1 and CycA-1 of Acidithiobacillus spp. and homologous cytochromes of Thiobacillus prosperus V6. The protein sequences were aligned using ClustalW (175 positions). The phylogeny was inferred by the Maximum Likelihood method based on the Whelan and Goldman model [157] [file pone.0146832.s004.pdf]
