# Supplementary material for: Genome Analysis of the Biotechnologically Relevant Acidophilic Iron Oxidising Strain JA12 Indicates Phylogenetic and Metabolic Diversity within the Novel Genus “Ferrovum”
Source: PLoS One. 2016 Jan 25;11(1):e0146832. doi: 10.1371/journal.pone.0146832 (PMC4725956; doi:10.1371/journal.pone.0146832)
Supplement: S5 Fig — The loci in the “Ferrovum” strain JA12 genome encoding proteins of the VirB/D4 type IV secretion system were compared with the locus in Agrobacterium tumefaciens. (A) The virB-operon in A. tumefaciens is based on [124]. (B) The trb-genes in strain JA12 are homologous to the vir-genes in A. tumefaciens as indicated in parentheses. Genes coloured in orange, grey, green or black are predicted to encode proteins of the VirB/D4 type IV secretion system, proteins presumably not related to the VirB/D4 system, mobile genetic elements (integrase, transposase) or hypothetical proteins, respectively. (PDF) [file pone.0146832.s005.pdf]

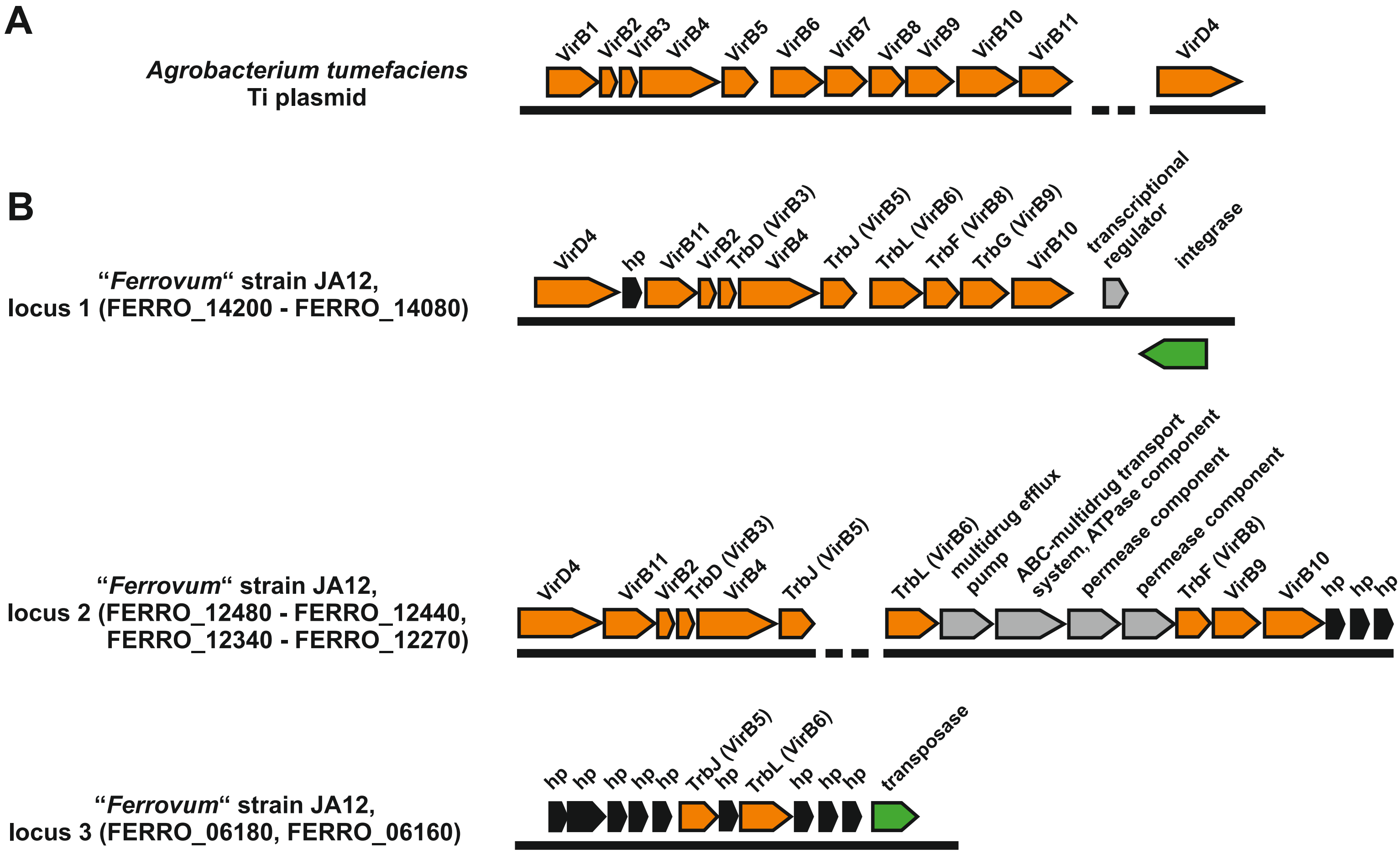
A
Agrobacterium tumefaciens
Ti plasmid
VirB1
VirB2
VirB3
VirB4
VirB5
VirB6
VirB7
VirB8
VirB9
VirB10
VirB11
VirD4
B
“Ferrovum“ strain JA12,
locus 1 (FERRO_14200 - FERRO_14080)
VirD4
hp
VirB11
VirB2
TrbD (VirB3)
VirB4
TrbJ (VirB5)
TrbL (VirB6)
TrbF (VirB8)
TrbG (VirB9)
VirB10
transcriptional regulator
integrase
“Ferrovum“ strain JA12,
locus 2 (FERRO_12480 - FERRO_12440,
FERRO_12340 - FERRO_12270)
VirD4
VirB11
VirB2
TrbD (VirB3)
VirB4
TrbJ (VirB5)
TrbL (VirB6)
multidrug efflux pump
ABC-multidrug transport system, ATPase component
permease component
permease component
TrbF (VirB8)
VirB9
VirB10
hp
hp
hp
“Ferrovum“ strain JA12,
locus 3 (FERRO_06180, FERRO_06160)
hp
hp
hp
hp
hp
TrbJ (VirB5)
hp
TrbL (VirB6)
hp
hp
hp
transposase
