# Supplementary material for: Genome Analysis of the Biotechnologically Relevant Acidophilic Iron Oxidising Strain JA12 Indicates Phylogenetic and Metabolic Diversity within the Novel Genus “Ferrovum”
Source: PLoS One. 2016 Jan 25;11(1):e0146832. doi: 10.1371/journal.pone.0146832 (PMC4725956; doi:10.1371/journal.pone.0146832)
Supplement: S1 Table — The protein-coding genes were assigned to the COG classes via the IMG/ER [141] prediction pipeline (17 February 2014). The percentage is based on the total number of protein-coding genes assigned to the COG categories (1,462). (DOCX) [file pone.0146832.s006.docx]

| **Code** | **Value** | **Percentage [%]** | **Description** |
| --- | --- | --- | --- |
| J | 171 | 10.59 | Translation, ribosomal structure and biogenesis |
| A | 1 | 0.06 | RNA processing and modification |
| K | 71 | 4.4 | Transcription |
| L | 77 | 4.77 | Replication, recombination and repair |
| B | 2 | 0.12 | Chromatin structure and dynamics |
| D | 31 | 1.92 | Cell cycle control, cell division, chromosome partitioning |
| V | 45 | 2.79 | Defense mechanisms |
| T | 62 | 3.84 | Signal transduction mechanisms |
| M | 147 | 9.1 | Cell wall/membrane biogenesis |
| N | 11 | 0.68 | Cell motility |
| U | 56 | 3.47 | Intracellular trafficking and secretion |
| O | 87 | 5.39 | Posttranslational modification, protein turnover, chaperones |
| C | 135 | 8.36 | Energy production and conversion |
| G | 66 | 4.09 | Carbohydrate transport and metabolism |
| E | 147 | 9.1 | Amino acid transport and metabolism |
| F | 49 | 3.03 | Nucleotide transport and metabolism |
| H | 107 | 6.63 | Coenzyme transport and metabolism |
| I | 73 | 4.52 | Lipid transport and metabolism |
| P | 69 | 4.27 | Inorganic ion transport and metabolism |
| Q | 26 | 1.61 | Secondary metabolites biosynthesis, transport and catabolism |
| R | 86 | 5.33 | General function prediction only |
| S | 71 | 4.4 | Function unknown |
| - | 547 | 27.23 | Not in COGs |
