# Supplementary material for: Genome Analysis of the Biotechnologically Relevant Acidophilic Iron Oxidising Strain JA12 Indicates Phylogenetic and Metabolic Diversity within the Novel Genus “Ferrovum”
Source: PLoS One. 2016 Jan 25;11(1):e0146832. doi: 10.1371/journal.pone.0146832 (PMC4725956; doi:10.1371/journal.pone.0146832)
Supplement: S4 Table — (DOCX) [file pone.0146832.s009.docx]

| **Name of the microorganism** | **Nucleotide Accession Number** |
| --- | --- |
| *Acidithiobacillus caldus* SM-1 | NC_015850 |
| *Acidithiobacillus caldus* SM-1 | NC_015850 |
| *Acidithiobacillus ferrivorans* SS3 | NC_015942 |
| *Acidithiobacillus ferrooxidans* ATCC 23270 | NC_011761 |
| *Alcanivorax borkumensis* SK2 | NC_008260 |
| *Cupriavidus basilensis* OR16 | NZ_AHJE01000000 |
| “*Ferrovum myxofaciens*” P3G | NZ_JPOQ00000000 |
| “*Ferrovum*”-like population FKB7 | 4565632.3 (MG-RAST) |
| *Gallionella capsiferriformans* ES-2 | NC_014394 |
| *Mariprofundus ferrooxydans* PV-1 | NZ_AATS00000000 |
| *Nitrosospira multiformis* ATCC 25196 | NC_007614 |
| *Prochlorococcus marinus* AS9601 | NC_008816 |
| *Pseudogulbenkiania ferrooxidans* 2002 | NZ_ACIS00000000 |
| *Rhodomicrobium vannielii* ATCC 17100 | NC_014664 |
| *Rhodopseudomonas palustris* TIE-1 | NC_011004 |
| *Rhodopseudomonas palustris* TIE-1 | NC_011004 |
| *Sideroxydans lithotrophicus* ES-1 | NC_013959 |
| *Thiobacillus denitrificans* ATCC 25259 | NC_007404 |
| *Thiobacillus prosperus V6* | EU653292 |
